# Hierarchically porous Co–N–C catalysts from bimetallic Zn/Co zeolitic imidazolate frameworks for selective CO_2_ electroreduction to CO

**DOI:** 10.3389/fchem.2026.1940515

**Published:** 2026-09-17

**Authors:** Zhaoyu Meng

**Affiliations:** School of Materials Science and Engineering, Nanyang Technological University, Singapore, Singapore

**Keywords:** Co–N_x_ sites, Co–N–C, electrochemical CO_2_ reduction, hierarchical porous carbon, metal–organic frameworks, zeolitic imidazolate framework

## Abstract

The conversion of CO_2_ to CO using electrochemistry requires the use of earth abundant catalysts with high selectivity, rapid reaction rate, and durability. In this work, hierarchically porous Co–N–C catalysts were synthesized via co-pyrolysis of a bimetallic zeolitic imidazolate framework (Zn/Co) and the dicyandiamide (DCD) followed by acid etching. By systematically adjusting the pyrolysis temperature (800 °C–1,000 °C) and the Co feed ratio (5–20 mol%), volcano-type activity relationships are found, which are controlled by the interplay of the three factors graphitization, nitrogen retention, and the density of Co–N_x_ sites. The optimized catalyst CoNC-900 achieves a Faradaic efficiency of CO of 94.9% ± 1.0% at −0.75 V vs. RHE in CO_2_-saturated 0.5 M KHCO_3_ with a CO partial current density of 16.0 mA cm^−2^, a lower bound turnover frequency of ≥0.33 s^−1^ and ≈11% current decay FE_CO retained above 92% over 24 h. The observed selectivity can be rationalized, as the density functional theory calculations indicate that the barrier of the rate-determining step, namely the formation of COOH is lowered on the Co–N_4_ moiety (0.42 eV, whereas ≥1.79 eV on the metal-free nitrogen sites). A transferable design rule is developed for CO_2_ electrolysis that relates bimetallic MOF precursor chemistry, hierarchical porosity, and M–N_x_ site engineering.

## Graphical Abstract

**Figure:**
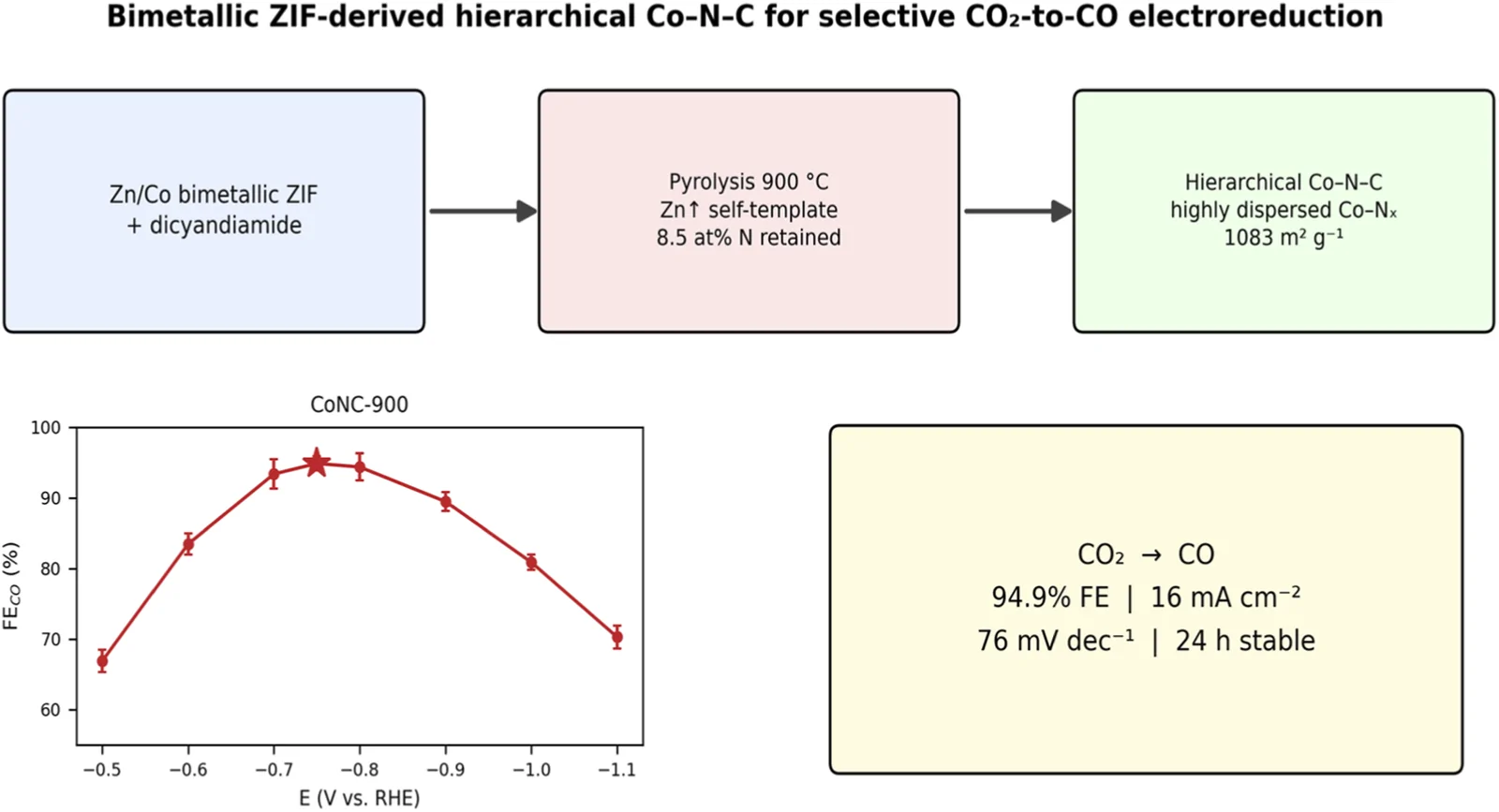

## Highlights

- Bimetallic Zn/Co-ZIF co-pyrolysis with dicyandiamide yields hierarchical Co–N–C.
- Zn acts as diluent and self-sacrificial porogen; DCD retains 8.5 at% N at 900 °C.
- CoNC-900 reaches 94.9% FE_CO and 16.0 mA cm^−2^ j_CO at −0.75 V vs. RHE.
- Volcano trends resolve roles of graphitization, N retention, and Co–N_x_ density.
- DFT: Co–N_4_ lowers the *COOH barrier to 0.42 eV, far below metal-free N sites.

## 1 Introduction

With the ever increasing level of CO_2_ in the atmosphere reaching 420 ppm due to anthropogenic emission exceeding 37 Gt yr^−1^, scalable carbon-capture-and-utilisation (CCU) technologies are considered as a scientific and social priority (Friedlingstein et al., 2023; IPCC, 2023). The electrochemical CO_2_ reduction reaction (CO_2_RR) using renewable electricity is particularly exciting due to several benefits: it is performed at ambient conditions of pressure and temperature, fits into intermittent renewable electricity, and turns a waste greenhouse gas into useful chemicals and fuels (Birdja et al., 2019; Nitopi et al., 2019). The field has traversed a wide landscape of CO_2_RR products ever since the initial product distribution surveys by Hori and co-workers for products with two electrons, including CO and formate, two-carbon products, including methanol and methane, and the much sought-after multi-carbon products resulting from C–C coupling on copper (Hori, 2008; Kuhl et al., 2012). Among these, the two-electron reduction of CO_2_ to CO is the most developed target, since CO is an important intermediate for Fischer–Tropsch synthesis and hydroformylation, and the shallow two-electron pathway avoids the harsh selectivity issues with the deeper reduction manifolds (Gao et al., 2019; Kibria et al., 2019). Electrocatalytic valorization strategies of this kind now extend well beyond CO_2_: catalyst development for the electrocatalytic depolymerization of lignin, for example, illustrates the same broader shift toward using renewable electricity to convert abundant, carbon-rich feedstocks into value-added products (Li et al., 2026).

The major challenge in CO_2_RR is the ability to drive the thermodynamically stable linear CO_2_ molecule to an intermediate bent CO_2_ radical anion (CO_2_R·) whose one-electron reduction potential is close to −1.9 V vs. SHE, while simultaneously preventing the kinetically favored hydrogen evolution reaction (HER), which competes for protons and electrons throughout the entire potential range relevant to CO_2_RR in aqueous media (Burdyny and Smith, 2019; Nitopi et al., 2019). Noble metals (e.g., Au and Ag) can catalyze the conversion of CO_2_ to CO with high selectivity, but they are very rare and expensive for terawatt-scale application (Lu and Jiao, 2016). In this context, atomically dispersed metal–nitrogen–carbon (M–N–C) catalysts represent a flurry of earth-abundant materials, where individual M–N_x_ units, whose structure closely mimics that of molecular metal–macrocycle complexes, form well-defined active sites and the surrounding sp^2^ carbon matrix offers electrical conductivity and mechanical strength (Ju et al., 2017; Pan F. et al., 2018; Zhao et al., 2017). Metal-free heteroatom doped carbons are also capable of reducing CO_2_, but are usually exploited with media engineering for desired selectivity (Kumar et al., 2013). In this family, single-atom Ni–N_x_ and Fe–N_x_ catalysts have been developed to exhibit high CO selectivity of nearly 100% (Ju et al., 2017; Li et al., 2017; Yang H. B. et al., 2018; Zhao et al., 2017) and Co-based catalysts have garnered a large amount of interest because the Co center exhibits a favorable balance between COOH stabilization and CO desorption, with several Co–N_x_ catalysts, such as the celebrated Co–N_5_ site anchored on hollow N-doped carbon spheres, seeing CO selectivity reach well beyond 90% over wide potential regions (Pan Y. et al., 2018; Wang et al., 2018) and the local-environment engineering of Co sites advancing CO selectivity and durability (Pei et al., 2024).

Notwithstanding this improvement, the activity of the M–N–C powder is often more strongly influenced by external (structure-related) factors. Three interlocked bottlenecks recur throughout the literature. First, the accessible density of active sites is typically not high, since a significant percentage of M–N_x_ moieties is shielded from the contacting electrolyte by dense microporous carbon (Varela et al., 2018). Second, at technologically relevant current densities, the mass transport of the sparingly soluble CO_2_ (≈33 mM in aqueous electrolyte at 1 atm and 25 °C) becomes rate limiting, leading to a local depletion of CO_2_, increase in the local pH and consequently a significant increase in HER (Burdyny and Smith, 2019; Weekes et al., 2018). Third, the high-temperature carbonization process that makes the carbon conductive also results in significant loss of nitrogen functionalities, which not only reduces the Co-coordinating environment, but also the metal-free nitrogen sites that help the adsorption of CO_2_ (Ju et al., 2017; Pels et al., 1995). Design requirements for a successful catalyst would thus include site density, hierarchical mass transport and nitrogen capture on one platform which is synthetically accessible.

Metal–organic frameworks (MOFs), and zeolitic imidazolate frameworks (ZIFs) in particular, are ideally suited to this challenge (Furukawa et al., 2013; Liu et al., 2008; Park et al., 2006). ZIF-derived carbons have been a workhorse of electrocatalysis since the self-templating of a MOF to porous carbon was demonstrated by Liu, Xu and co-workers (Liu et al., 2008). The nitrogen-rich imidazolate linker of ZIF-8 provides an intrinsic nitrogen reservoir, volatilization of the Zn nodes above their boiling point (907 °C) opens porosity without any external hard template or corrosive etchant, and the second less volatile metal introduced in the framework can be spatially dispersed by the surrounding Zn/imidazolate matrix, favoring disperse atomic dispersion of the M–N_x_ sites over aggregation to form inactive nanoparticles (Gong et al., 2020; Lu et al., 2020; Park et al., 2006; Xia et al., 2015). This bimetallic Zn/Co-ZIF strategy is particularly appealing because the porogenic function of Zn and catalytic function of Co are integrated into one atomic precursor which is then encoded to form the pore structure and active sites prior to the pyrolysis process (Gong et al., 2020; Lu et al., 2020). The general strategy of pairing two metals with distinct functional roles within one precursor is not unique to CO_2_RR catalysts: bimetallic Fe/Zn systems, for instance, have recently been used to engineer lignin-based Fenton-like catalysts for water remediation, underscoring the broader applicability of rationally paired bimetallic active-site design (Liu et al., 2026).

Nevertheless, two design variables remain insufficiently disentangled. The pyrolysis temperature simultaneously controls graphitization (and hence conductivity), nitrogen retention, and metal speciation, producing non-monotonic activity trends that are widely reported but rarely deconvoluted with a complete, self-consistent set of structural, textural, electronic, and kinetic descriptors on a single sample series. Likewise, the metal loading governs the trade-off between active-site density and aggregation, yet its optimum is seldom pinned down against a Co-free control prepared by an otherwise identical route. In this work, we combine a bimetallic Zn/Co-ZIF precursor with DCD as an auxiliary nitrogen source in a simple ball-mill/co-pyrolysis route, followed by acid etching, to obtain hierarchically porous Co–N–C catalysts (denoted CoNC-T, where T is the pyrolysis temperature). The DCD co-precursor sustains a nitrogen content of 8.5 at% even after carbonization at 900 °C—roughly three times the value typical of DCD-free ZIF pyrolysis—while Zn evaporation and gas evolution build a bimodal micro/mesopore network with a BET surface area of 1,083 m^2^ g^−1^ and pronounced CO_2_ affinity. By systematically varying the pyrolysis temperature (800, 900, 1,000 °C) and the Co feed ratio (5, 10, 20 mol%), and by benchmarking against a Co-free N-doped carbon (NC-900) and commercial carbon black (CB-Vulcan), we resolve two clear volcano relationships and identify CoNC-900 as the optimum, achieving FE_CO = 94.9% and j_CO = 16.0 mA cm^−2^ at −0.75 V vs. RHE with a 76 mV dec^−−1^ Tafel slope and 24 h stability. Computational DFT studies on different models (Co–N_4_, pyridinic-N, graphitic-N, pristine-carbon) reveal that COOH formation is the rate-determining step on all sites and that only Co–N_4_ brings the barrier down into a catalytically relevant window, thereby providing a mechanistic picture that links precursor design, site speciation, and CO selectivity. The novelty of this study does not reside in the precursor family itself, Zn/Co-ZIF pyrolysis has been well established, but in the complete and self-consistent deconvolution that is provided: (1) fitted kinetics, (2) site-normalized activity, and (3) DFT on a common footing across a seven-sample matrix (4) temperature, (5) metal loading, and (6) metal-free and (7) non-templated controls. Overall concept of the synthetic approach is depicted in Figure 1.

**FIGURE 1:**
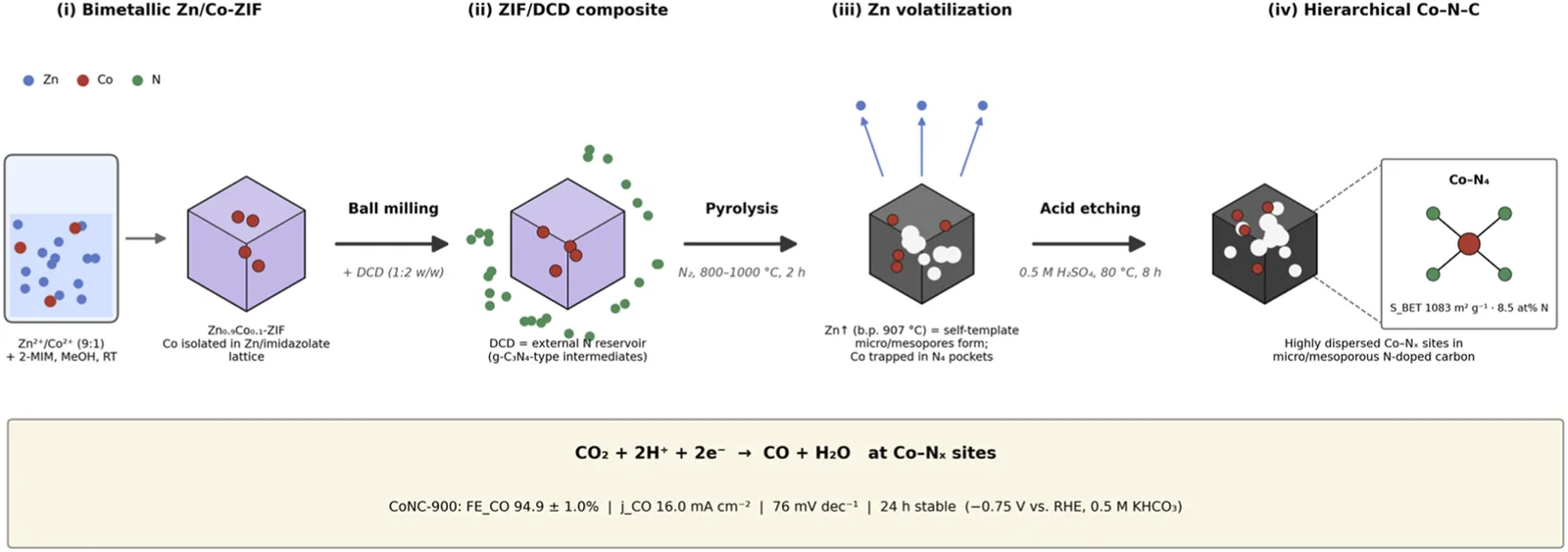
Illustrated synthesis route to hierarchically porous Co–N–C catalysts. (i) Room-temperature crystallization of a bimetallic Zn_0_._9_Co_0_._1_-ZIF, in which Co^2+^ is diluted within the Zn/imidazolate lattice; (ii) ball-milling with dicyandiamide (DCD) as an external nitrogen reservoir; (iii) pyrolysis under N_2_, during which Zn volatilizes (b.p. 907 °C) as a self-sacrificial template, generating micro/mesoporosity while Co is trapped in nitrogen-rich pockets; (iv) acid etching removes residual Zn and acid-soluble Co, leaving highly dispersed Co–N_x_ sites (Co–N_4_ model in inset) in a micro/mesoporous N-doped carbon.

## 2 Experimental section

### 2.1 Chemicals and materials

Zinc nitrate hexahydrate (Zn(NO_3_)_2_·6H_2_O, AR, ≥99%), cobalt nitrate hexahydrate (Co(NO_3_)_2_·6H_2_O, AR, ≥99%), 2-methylimidazole (2-MIM, 98%), and KHCO_3_ (AR, ≥99.5%) were obtained from Aladdin. Dicyandiamide (DCD, ≥99%) was obtained from the Macklin. Methanol (AR, ≥99.5%), ethanol (AR, ≥99.7%), and sulfuric acid (98%) were supplied by Sinopharm. Nafion solution (5 wt%, D520) was bought from DuPont and Vulcan XC-72R carbon black from Cabot. Research-grade CO_2_ and N_2_ (both 99.999%) were used for saturation and control experiments. All chemicals were used as received and ultrapure water (18.2 MΩ cm) was used for all purposes.

### 2.2 Synthesis of bimetallic Zn/Co-ZIF precursors

In a representative preparation of Zn_0_._9_Co_0_._1_-ZIF, Zn(NO_3_)_2_·6H_2_O (2.376 g, 8.0 mmol) and Co(NO_3_)_2_·6H_2_O (0.291 g, 1.0 mmol) were dissolved in methanol (100 mL) under magnetic stirring (solution A), while 2-MIM (6.571 g, 80.0 mmol) was dissolved in methanol (100 mL) (solution B). Solution B was added quickly to solution A with vigorous stirring (1,000 rpm) and stirred for 10 min and then allowed to age undisturbed at room temperature for 24 h. The purple-blue precipitate was collected by centrifugation (8,000 rpm for 10 min), washed with fresh methanol (3 × 50 mL), and dried under vacuum at 60 °C for 12 h until a constant mass was obtained (≈3.5 g). The molar ratio of linker to metal was kept at 8.9:1 so as to have complete coordination. Precursors with 5 mol% Co (CoNC-900-L; 8.5 mmol Zn + 0.5 mmol Co) and 20 mol% Co (CoNC-900-H; 7.0 mmol Zn + 2.0 mmol Co) were prepared in the same way, as was the Co-free ZIF-8 precursor (NC-900; 9.0 mmol Zn), by adjusting the Zn/Co salt ratio while keeping the total amount of metal salt constant.

### 2.3 Preparation of CoNC-T catalysts

The as-prepared ZIF powder was mixed with DCD at a ratio of mass of powder to mass of DCD as 1:2 and homogenized by planetary ball milling (400 rpm, 2 h; ZrO_2_ balls, ball-to-powder mass ratio 20:1). The homogeneous mixture was placed in a horizontally placed tube furnace in an alumina boat and treated with N2 (100 mL min^−1^) for 30 min, then heated at a rate of 2 °C min^−1^ to the temperature desired (800, 900, or 1,000 °C), followed by a 2-h treatment at the target temperature and then cooled naturally to room temperature under N_2_ purge. The pyrolyzed powders were etched in 0.5 M H_2_SO_4_ (100 mL) at 80 °C for 8 h to remove unstable/uncoordinated Co species, residual Zn, and soluble byproducts, filtered through a 0.45 μm PVDF membrane, washed with deionized water to pH ≥ 6, and dried at 80 °C for 12 h (typical yield 40%–55%). The samples are denoted CoNC-800, CoNC-900, and CoNC-1000 (10 mol% Co feed; temperature series), CoNC-900-L (5 mol% Co) and CoNC-900-H (20 mol% Co) (loading series), and NC-900 (Co-free control). Vulcan XC-72R (CB-Vulcan) was used as received as a baseline.

### 2.4 Materials characterization

Powder X-ray diffraction (XRD) patterns were recorded on a Rigaku SmartLab 9 kW diffractometer (Cu Kα, λ = 1.5406 Å) over 5°–80° 2θ. Raman spectra were collected on a Horiba LabRAM HR Evolution spectrometer (532 nm, 5 mW). N_2_ adsorption–desorption isotherms were measured at 77 K on a Micromeritics ASAP 2460 after degassing at 200 °C for 12 h under vacuum; specific surface areas were obtained by the BET method (P/P_0_ = 0.05–0.30), and pore-size distributions by NLDFT using a carbon slit-pore model. CO_2_ adsorption isotherms were measured at 273 and 298 K on the same instrument. X-ray photoelectron spectroscopy (XPS) was performed on a Thermo Fisher ESCALAB 250Xi (monochromated Al Kα, 1,486.6 eV); binding energies were charge-corrected to C 1s = 284.8 eV, and high-resolution N 1s and Co 2p spectra were deconvoluted with a Shirley background and Gaussian–Lorentzian (GL30) line shapes in the Avantage software. Bulk metal contents were determined by ICP-OES (Agilent 5110) after microwave-assisted aqua-regia digestion. Morphology and elemental distribution were examined by field-emission SEM and TEM equipped with an Oxford X-MaxN 80 EDS detector (15 kV).

### 2.5 Electrochemical measurements

Catalyst inks were prepared by ultrasonicating 2.0 mg of catalyst in a mixture of ethanol (380 μL), water (100 μL), and 5 wt% Nafion (20 μL) in an ice-water bath for 30 min. The ink was drop-cast onto a polished glassy carbon electrode (d = 5 mm, geometric area 0.196 cm^2^) to a mass loading of 0.51 mg cm^−2^. All measurements were performed on a CHI 760E workstation in a gas-tight two-compartment H-cell separated by a pretreated Nafion 117 membrane, with 35 mL of 0.5 M KHCO_3_ in each compartment, an Ag/AgCl (3 M KCl) reference electrode, and a Pt-wire counter electrode. The electrolyte was saturated with CO_2_ (20 mL min^−1^, 30 min; pH 6.8) before and continuously during electrolysis; N_2_-saturated electrolyte was used for controls. Potentials were converted to the RHE scale as E(RHE) = E(Ag/AgCl) + 0.210 + 0.0592 × pH (+0.612 V, verified against a H_2_-purged Pt electrode). iR compensation was applied at 85% during measurement, with the residual 15% corrected in post-processing using the high-frequency EIS intercept (7–10 Ω). Linear sweep voltammetry (LSV) was recorded at 5 mV s^−1^ from +0.2 to −1.1 V vs. RHE after 20 conditioning CV cycles. Chronoamperometry for product quantification was carried out for 30 min at each potential from −0.50 to −1.10 V vs. RHE. Tafel slopes were obtained by least-squares regression of overpotential (relative to E°(CO_2_/CO) = −0.11 V vs. RHE) against log j_CO over the kinetically controlled region, defined as the potentials preceding the onset of curvature in the Tafel plot and at which j_CO remained below 30% of the diffusion-limited current estimated from the LSV plateau; the exact window, number of points, and R^2^ for each sample are listed in Supplementary Table S6. Electrochemical impedance spectroscopy (EIS) was measured at −0.70 V vs. RHE (5 mV rms, 100 kHz–10 mHz) and fitted to an Rs + Q/(Rct + Zw) equivalent circuit (χ^2^ < 0.01) in ZView. The double-layer capacitance (C_dl) was obtained from CVs at 20–100 mV s^−1^ in the non-Faradaic window (0.15–0.25 V vs. RHE), and the electrochemically active surface area (ECSA) was estimated as C_dl/C_s with C_s = 0.040 mF cm^−2^, a conventional reference value for carbon surfaces in aqueous electrolyte (McCrory et al., 2013); because absolute ECSA on heteroatom-doped porous carbons carries systematic uncertainty from incomplete pore wetting and ion accessibility, ECSA is used here only comparatively across an identically prepared series; C_dl values were obtained by ordinary least-squares regression of Δj/2 at 0.20 V against scan rate (R^2^ ≥ 0.999 for all samples). The stability of CoNC-900 was assessed by 24 h chronoamperometry at −0.75 V vs. RHE with FE_CO sampled every 4 h.

### 2.6 Product quantification

Gaseous products were quantified by on-line gas chromatography (Agilent 7890B; TCD for H_2_, CO, and CO_2_ and FID with a methanizer for trace hydrocarbons; Carboxen-1000 column; Ar carrier, 30 mL min^−1^) using a six-point calibration (50–10,000 ppm; detection limits: CO 10 ppm, H_2_ 5 ppm, CH_4_ 2 ppm). Headspace samples (1 mL) were taken at t = 25 min of each potentiostatic step. Liquid products were analyzed by ^1^H NMR (Bruker AVANCE 400 MHz, WATERGATE water suppression, 512 scans) with DMSO as internal standard; only trace formate (0.032 ± 0.005 mM, FE < 1.5%) was detected, with methanol, ethanol, and acetate all below detection limits. Faradaic efficiencies were calculated as FE = nFN_product/Q_total (n = 2 for CO and H_2_), and all values are means ± standard deviation of three independently prepared electrodes (n = 3, separate ink depositions on freshly polished glassy carbon); total FE closed to 99%–100% at every potential.

### 2.7 DFT calculations

Spin-polarized DFT calculations were performed with VASP 6.3.2 using the PBE functional with D3(BJ) dispersion correction and PAW pseudopotentials (plane-wave cutoff 450 eV; Γ-centered 3 × 3 × 1 k-mesh; electronic convergence 10^–5^ eV; force convergence 0.02 eV Å^-1^) (Blöchl, 1994; Grimme et al., 2011; Kresse and Furthmüller, 1996; Perdew et al., 1996). Implicit aqueous solvation was described by the VASPsol model (ε_r = 78.4) (Mathew et al., 2014). Four model sites were constructed on a 5 × 5 graphene supercell with 15 Å vacuum: a Co–N_4_ moiety (Co substituting C, coordinated by four pyridinic N), an edge pyridinic-N site, a bulk graphitic-N site, and pristine graphene. Free energies of the CO_2_ → COOH → CO → CO(g) pathway were evaluated with the computational hydrogen electrode (CHE) model, G = E_DFT + ZPE − TS + ΔG_solv, with potential dependence G(U) = G(0) + neU (Nørskov et al., 2004). Zero-point energies and entropies were obtained from vibrational analysis (finite displacements, 0.01 Å) and NIST-JANAF tables at 298 K. Additional characterization and control data (XPS survey spectra, capacitance voltammograms, 298 K CO_2_ isotherms, ^1^H NMR, GC calibration, H_2_ selectivity, and full fitting parameters) are provided in the Supplementary Material (Supplementary Figures S1–S8; Supplementary Tables S1–S8).

## 3 Results and discussion

### 3.1 Synthesis rationale, morphology, and composition

The design concept (Figure 1) makes use of the bimetallic nature of the precursor. At room-temperature crystallization, Co^2+^ is *in situ* substitutionally incorporated into the ZIF-8 lattice, which results in each Co center being surrounded by a Zn/imidazolate lattice and spatially separated from other Co centers. During heating, DCD decomposes via intermediates of g-C_3_N_4_ type that are produced by ball milling the DCD, and produces reactive C–N species that replenish the nitrogen released from the carbonizing framework. The Zn nodes evaporate above 907 °C, causing the simultaneous creation of mesoporosity and leaving behind vacant N_4_ pockets where the less-volatile Co is trapped as highly dispersed Co–N_x_ sites; acid washing removes acid-soluble Co species and any residual Zn, leaving behind robustly coordinated Co in the carbon.

SEM images (Figures 2a–c) show faceted polyhedral carbon particles with well-defined edges after pyrolysis at 800 °C and 900 °C, whereas at 1,000 °C the edges become rounded and inter-particle fusion occurs. The progressive loss of edge definition with increasing pyrolysis temperature is consistent with the progressive densification and partial sintering reported for MOF-derived carbons at elevated pyrolysis temperatures (Xia et al., 2015). TEM images of CoNC-900 and NC-900 (Figures 2d, e) show a disordered, porous carbon texture without detectable Co nanoparticles or graphitic core–shell (Co@C) features, while the EDS elemental mapping (Figure 2f) shows the uniform distribution of C, N, and Co throughout the particle without any clusters of Co-rich particles. Quantitatively (Table 1), ICP-OES gives bulk Co contents of 3.82, 2.91, and 2.38 wt% for CoNC-800, CoNC-900, and CoNC-1000, respectively, and 1.48 and 5.83 wt% for CoNC-900-L and CoNC-900-H, tracking the Co feed ratio. Residual Zn falls from 2.15 wt% (CoNC-800) to 0.34 wt% (CoNC-900) and 0.11 wt% (CoNC-1000), showing that Zn volatilization is incomplete below the Zn boiling point and essentially complete above it; the surface Zn 2p XPS signal follows the same trend (1.7 → 0.4 → 0.3 at%). Residual Zn is present at a comparable level in the Co-free NC-900 (0.29 wt%), which provides an internal control for any contribution of Zn–N_x_ sites to the CO activity (Section 3.5). The observed levels of Fe and Ni impurities were less than 0.01 wt% (excluding adventitious-metal contributions to the activity observed) in all samples, which has been shown to be an important control due to the sensitivity of CO_2_RR to trace transition metals (Ju et al., 2017; Yang H. B. et al., 2018).

**FIGURE 2:**
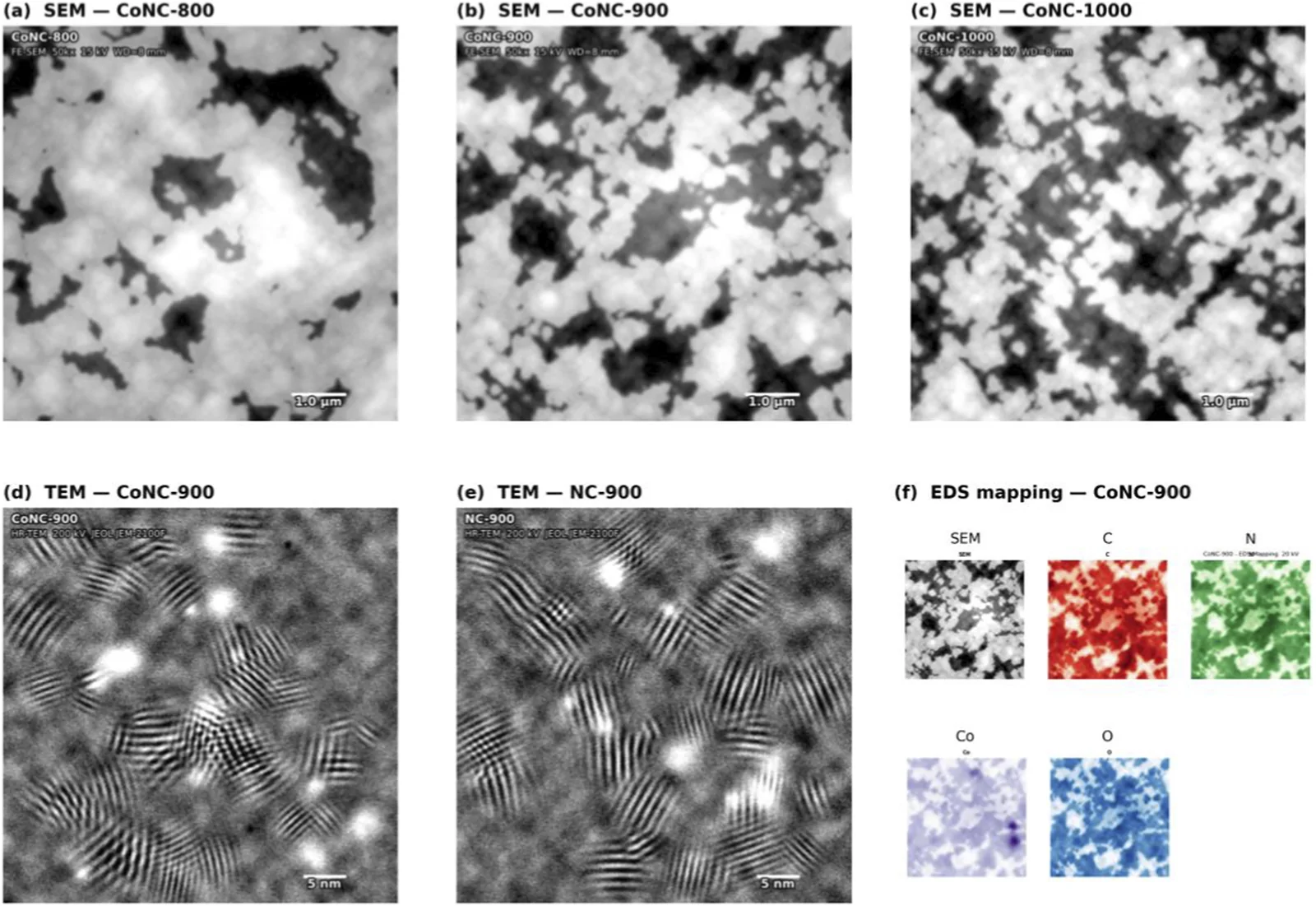
**(a–c)** SEM images of CoNC-800, CoNC-900, and CoNC-1000 showing faceted polyhedral carbon particles, with well-defined edges at 800 °C and 900 °C and edge rounding with inter-particle fusion at 1,000 °C (scale bars 1.0 μm). **(d,e)** TEM images of CoNC-900 and NC-900 showing a porous, disordered carbon texture without Co nanoparticles or core–shell features (scale bars 5 nm). **(f)** EDS elemental maps (SEM, C, N, Co, O) of CoNC-900 showing homogeneous C, N, and Co distribution.

**TABLE 1:** Physicochemical properties of the catalysts. BET surface area (S_BET), total/micro/meso pore volumes, Raman I_D/I_G, bulk Co and Zn contents (ICP-OES), and surface N content (XPS).

Sample	S_BET (m^2^ g^−1^)	V_total (cm^3^ g^−1^)	V_micro (cm^3^ g^−1^)	V_meso (cm^3^ g^−1^)	I_D/I_G	Co (wt%)	Zn (wt%)	N (at%)
CoNC-800	782	0.48	0.28	0.20	1.15	3.82	2.15	12.8
CoNC-900	1,083	0.72	0.38	0.34	1.05	2.91	0.34	8.5
CoNC-1000	1,198	0.85	0.35	0.50	0.97	2.38	0.11	5.2
CoNC-900-L	1,022	0.65	0.35	0.30	1.06	1.48	0.31	8.2
CoNC-900-H	948	0.62	0.32	0.30	1.04	5.83	0.37	8.8
NC-900	981	0.63	0.34	0.29	1.08	<0.01	0.29	9.1
CB-Vulcan	248	0.25	0.05	0.20	1.35	<0.01	<0.01	0.3

### 3.2 Crystal structure and degree of graphitization

The XRD patterns of all CoNC-T samples (Figure 3a) display only two broad reflections at ≈24° and ≈44° 2θ, assignable to the (002) and (10) planes of turbostratic carbon (Li et al., 2007; Warren, 1941). Because the (111) reflection of face-centred-cubic metallic Co (JCPDS 15-0806) falls at 44.2° and overlaps this region, the assignment was scrutinized in three ways. First, the ≈44° band is broad (FWHM 5°–7° 2θ) and centred at 43.0°–43.9°, whereas a crystalline Co phase would give a sharp reflection at 44.2°; second, the companion Co (200) and (220) reflections at 51.5° and 75.9° are absent in every Co-containing pattern (residual intensity ≤3% and ≤1% of the (002) band, respectively, indistinguishable from baseline noise); and third, the Co-free NC-900 exhibits an identical band at 43.7° with the same width, demonstrating that the feature originates from the carbon lattice. Likewise, no reflections of CoO or Co_3_O_4_ (e.g., 36.8°) are detected. The absence of these reflections indicates that the Co species are too dilute and/or too small to develop long-range order, consistent with a high degree of dispersion rather than crystalline nanoparticles. Scherrer analysis of the (002) reflection gives an interlayer spacing d_002_ decreasing from 3.89 Å (CoNC-800) to 3.65 Å (CoNC-900) and 3.45 Å (CoNC-1000), with the stacking height Lc increasing from 8.1 to 10.8 and 16.2 nm, i.e., from ≈21 to ≈30 and ≈47 stacked layers (Supplementary Table S7), a quantitative signature of progressive graphitization with pyrolysis temperature (Li et al., 2007). Raman spectra (Figure 3b) show the characteristic D (≈1,350 cm^−1^) and G (≈1,580 cm^−1^) bands (Ferrari and Robertson, 2000; Tuinstra and Koenig, 1970), with I_D/I_G decreasing monotonically from 1.15 (CoNC-800) to 1.05 (CoNC-900) and 0.97 (CoNC-1000). Using the excitation-wavelength-dependent Cançado relation for 532 nm (Cançado et al., 2006), the in-plane crystallite size La rises correspondingly from 16.7 to 18.3 and 19.8 nm (Supplementary Table S7). This progressive defect annealing at higher temperature increases electrical conductivity, as corroborated by the impedance data in Section 3.6, but comes at the expense of nitrogen retention (Section 3.4; Pels et al., 1995). CB-Vulcan exhibits the highest structural disorder (I_D/I_G = 1.35; La = 14.2 nm), consistent with its amorphous, non-templated origin, in line with the established interpretation of the D-band intensity as a measure of disorder in nanostructured carbons (Ferrari and Robertson, 2000; Tuinstra and Koenig, 1970). Pyrolysis temperature thus emerges as a direct and continuous handle on the electronic quality of the carbon framework.

**FIGURE 3:**
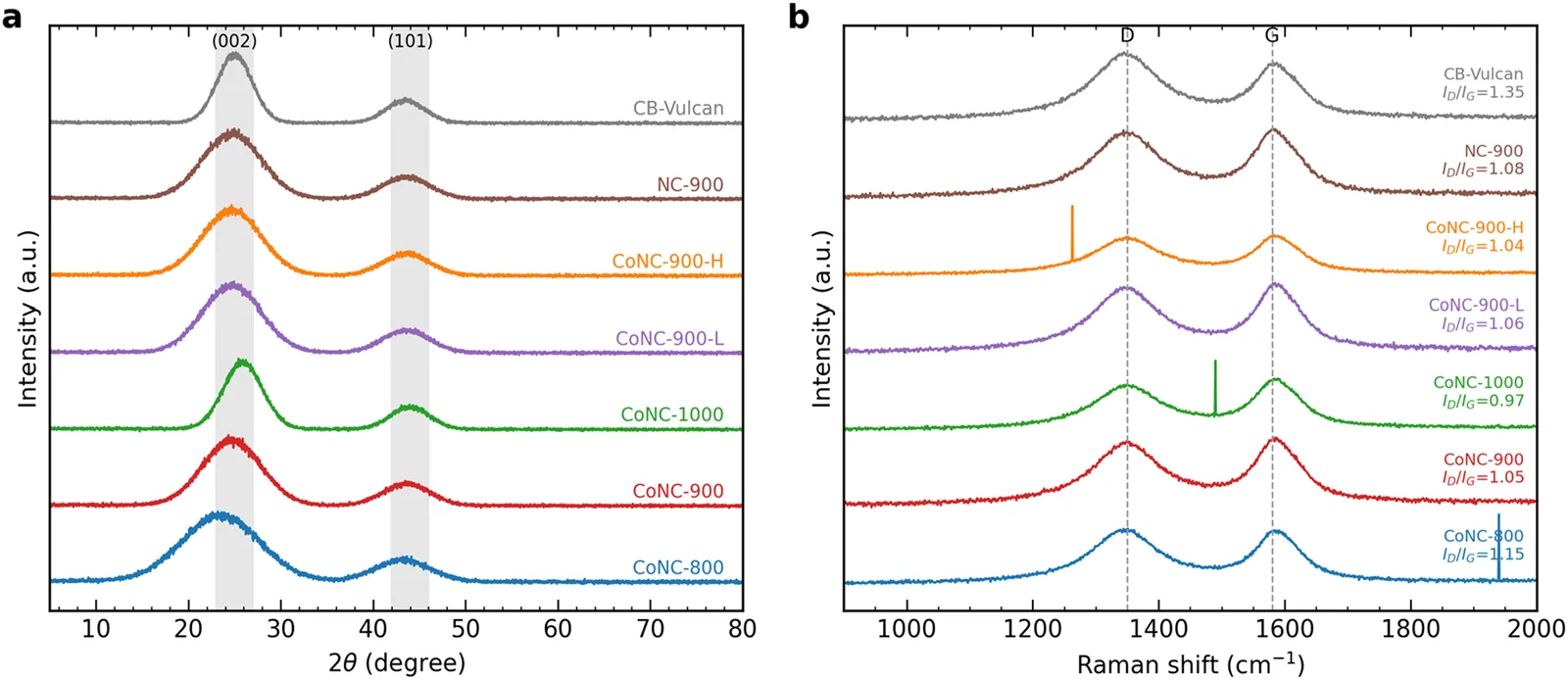
**(a)** XRD patterns and **(b)** Raman spectra of the seven catalysts. The broad ≈44° band is the carbon (10) reflection: it is present with identical width and position in the Co-free NC-900, and the Co (200) and (220) reflections at 51.5° and 75.9° are absent in all Co-containing samples. The monotonic decrease of I_D/I_G with pyrolysis temperature in **(b)** quantifies progressive graphitization (Lc, La in Supplementary Table S7).

### 3.3 Hierarchical porosity and CO_2_ uptake

The N_2_ physisorption isotherms of the CoNC-T samples (Figure 4a) combine steep low-pressure uptake—microporosity inherited from the sodalite cages of the ZIF precursor—with a type-H4 hysteresis loop at intermediate relative pressures, signalling mesoporosity created by Zn evaporation and gas evolution. The resulting texture is genuinely hierarchical, as required for efficient CO_2_ transport. The BET surface area rises from 782 m^2^ g^−1^ (CoNC-800) to 1,083 m^2^ g^−1^ (CoNC-900) and 1,198 m^2^ g^−1^ (CoNC-1000), closely tracking the extent of Zn removal, while the total pore volume grows from 0.48 to 0.72 and 0.85 cm^3^ g^−1^ and the mesopore fraction expands from 42% to 47% and 59% of the total pore volume (Table 1). NLDFT pore-size distributions (Figure 4b) confirm coexisting sub-2-nm micropores and 2–10 nm mesopores, with the mean pore width widening from 1.82 nm (800 °C) to 2.78 nm (1,000 °C). All ZIF-derived carbons vastly exceed CB-Vulcan (248 m^2^ g^−1^, essentially non-microporous), underscoring the templating role of the MOF precursor. The value of deliberately engineered multiscale porosity for mass transport and active-site accessibility is a recurring theme across electrocatalysis and adsorption science more broadly, from hierarchically structured biomass-derived aerogels built on a “brick-mortar-binder” design principle (Li et al., 2025) to phosphorus-doped transition-metal oxide nanosheets anchored on carbon microspheres for water splitting (Zhang et al., 2026).

**FIGURE 4:**
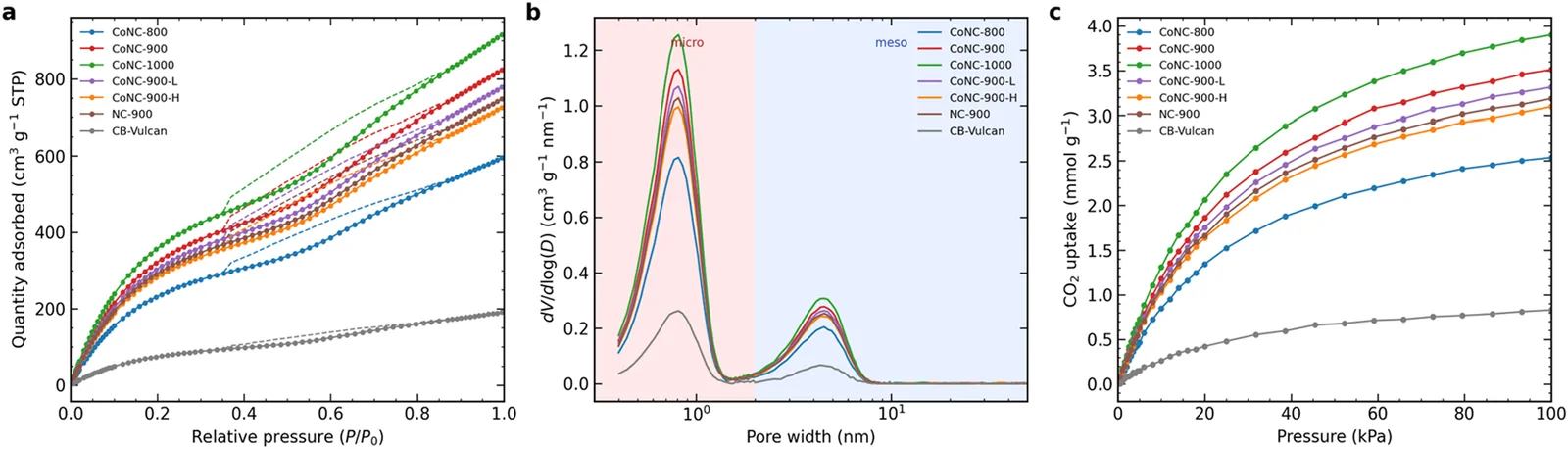
**(a)** N_2_ adsorption–desorption isotherms at 77 K (filled symbols, adsorption; dashed, desorption), **(b)** NLDFT pore-size distributions with micropore (<2 nm) and mesopore (2–50 nm) regions shaded, and **(c)** CO_2_ adsorption isotherms at 273 K for all catalysts.

The functional consequence of this porosity is evident in the CO_2_ adsorption isotherms at 273 K (Figure 4c), where the uptake at 100 kPa reaches 3.51 mmol g^−1^ for CoNC-900 and 3.90 mmol g^−1^ for CoNC-1000, versus 2.54 mmol g^−1^ for CoNC-800, 3.19 mmol g^−1^ for NC-900, and only 0.83 mmol g^−1^ for CB-Vulcan. The strong CO_2_ affinity of the ZIF-derived carbons arises from the synergy of narrow micropores that confine CO_2_ and abundant nitrogen functionalities that polarize the adsorbate, a combination well documented for nitrogen-doped porous carbons (Hao et al., 2010; Sevilla et al., 2011). This local CO_2_ enrichment near the active sites is expected to mitigate the mass-transport penalty imposed by the low CO_2_ solubility in aqueous electrolyte, and it distinguishes the hierarchical carbons from conventional microporous or macroporous supports that optimize only one length scale (Varela et al., 2018; Weekes et al., 2018).

### 3.4 Surface chemistry and Co speciation

XPS survey spectra confirm the presence of C, N, O, Co, and residual Zn in the CoNC samples. The surface nitrogen content decreases from 12.8 at% (CoNC-800) to 8.5 at% (CoNC-900) and 5.2 at% (CoNC-1000), quantifying the fundamental trade-off between graphitization and nitrogen retention. Notably, the DCD co-precursor keeps the nitrogen content at 900 °C well above values commonly reported for ZIF-derived carbons pyrolyzed at comparable temperatures without an auxiliary nitrogen source (Ju et al., 2017; Pels et al., 1995). High-resolution N 1s spectra (Figure 5a) were deconvoluted into five components: pyridinic-N (398.5 eV), Co–N_x_ (399.2–399.3 eV), pyrrolic-N (400.3 eV), graphitic-N (401.2 eV), and oxidized-N (403.5 eV). For CoNC-900, pyridinic-N (35.2%) and Co–N_x_ (18.5%) dominate the spectrum, whereas at 1,000 °C the Co–N_x_ fraction falls to 14.2% and graphitic-N grows to 35.8%, consistent with thermally driven conversion of edge/coordinating nitrogen into lattice nitrogen. This redistribution is central to the activity trends discussed below, because pyridinic-N and Co–N_x_ are the functionalities most closely associated with CO_2_ activation, while graphitic-N contributes mainly to conductivity (Ju et al., 2017; Ju et al., 2019; Kumar et al., 2013).

**FIGURE 5:**
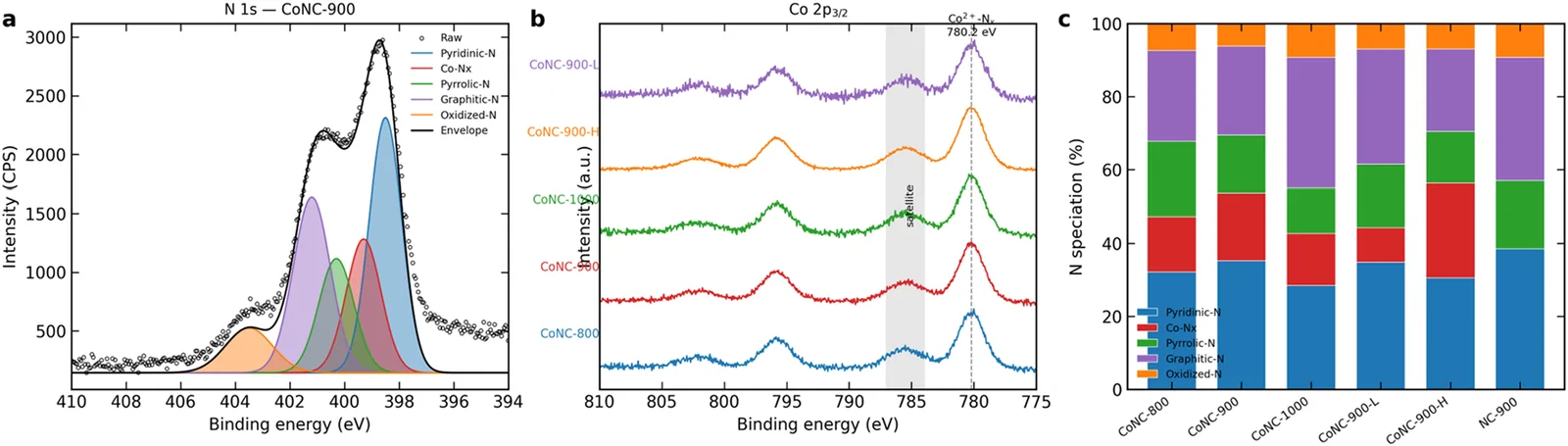
**(a)** Deconvoluted high-resolution N 1s spectrum of CoNC-900 showing pyridinic-N, Co–N_x_, pyrrolic-N, graphitic-N, and oxidized-N. **(b)** Co 2p_3_/_2_ spectra of the CoNC series, all centered at 780.2 eV (Co^2+^–N_x_) with satellite features and no metallic-Co component. **(c)** Evolution of N speciation with pyrolysis temperature and Co feed ratio.

The assignment of the 399 eV feature to Co–N_x_ is corroborated by three independent observations. First, the Co–N_x_ component scales with the Co feed ratio, rising from 9.5% (CoNC-900-L, 5 mol%) to 18.5% (CoNC-900, 10 mol%) and 25.8% (CoNC-900-H, 20 mol%). Second, it is entirely absent in the Co-free NC-900 control, whose N 1s spectrum resolves into only four components. Third, the N speciation evolution across the series (Figure 5c) tracks these compositional changes consistently. The Co 2p spectra of all CoNC samples (Figure 5b) were analysed quantitatively (Supplementary Table S8): the Co 2p_3_/_2_ main line lies at 780.0–780.3 eV (FWHM 2.4–2.5 eV) with a spin–orbit splitting of 15.5–16.1 eV and an intense shake-up satellite at 785.1–785.5 eV, i.e., 4.9–5.5 eV above the main line with a satellite-to-main intensity ratio of 0.34–0.38. This combination—main-line position, large satellite separation, and high satellite intensity—is the fingerprint of high-spin Co^2+^ in a nitrogen/oxygen coordination environment (Biesinger et al., 2011); metallic Co would give a narrow asymmetric line at 778.1–778.3 eV without a strong satellite, and Co_3_O_4_ would give a main line near 779.6 eV with a weak, distant satellite (≈9 eV) (Biesinger et al., 2011), neither of which is observed. Within the GL30 fitting, all intensity at 778.2 eV is accounted for by the low-binding-energy tail of the 780.2 eV component, and no separate metallic component is required. In ZIF-derived carbons, metallic Co encapsulated within graphitic shells (Co@C) is known to resist acid leaching (Deng et al., 2015), and its presence is therefore assessed here directly by diffraction, microscopy, and spectroscopy. The fcc-Co (111), (200), and (220) reflections expected at 44.2°, 51.5°, and 75.9° are absent from all patterns (Section 3.2), no core–shell contrast is observed by TEM (Figure 2d), and the Co 2p line shape shows no component at 778.1–778.3 eV. Taken together with the homogeneous Co distribution in EDS mapping and the scaling of the Co–N_x_ N 1s component with loading, these data indicate that the Co is predominantly present as highly dispersed Co–N_x_ coordination species embedded in the carbon matrix, and the Co–N_4_ moiety is adopted as the representative site model for the DFT analysis (Section 3.8).

### 3.5 Electrocatalytic CO_2_ reduction performance

LSV curves recorded in CO_2_- versus N_2_-saturated 0.5 M KHCO_3_ (Figure 6a; N_2_ controls for all seven samples in Supplementary Figure S8) reveal a clear anodic shift of the onset potential and markedly enhanced current under CO_2_ for all Co-containing samples: at −0.75 V, the N_2_-saturated current is only 15%–35% of the CO_2_-saturated current for the CoNC series (15% for CoNC-900), compared with 69% for NC-900 and 89% for CB-Vulcan, confirming that the CO_2_-dependent current arises from CO_2_ reduction on the Co-containing catalysts rather than from hydrogen evolution. CoNC-900 reaches total current densities of ≈−17 mA cm^−2^ at −0.75 V and ≈−50 mA cm^−2^ at −1.10 V vs. RHE (interpolated at the chronoamperometry potentials), substantially higher than NC-900 and CB-Vulcan under identical conditions. Potential-dependent Faradaic efficiencies (Figure 6b) show that CO and H_2_ together account for 99%–100% of the passed charge in all cases, with only trace formate (<1.5%) detected by ^1^H NMR, in line with the expectation that H-cell operation in neutral electrolyte favors gaseous products (Kibria et al., 2019; Pan Y. et al., 2018). CoNC-900 exhibits a broad selectivity plateau, with FE_CO exceeding 90% from −0.70 to −0.80 V and peaking at 94.9% ± 1.0% at −0.75 V vs. RHE, where the CO partial current density reaches 16.0 mA cm^−2^ (Figure 6c).

**FIGURE 6:**
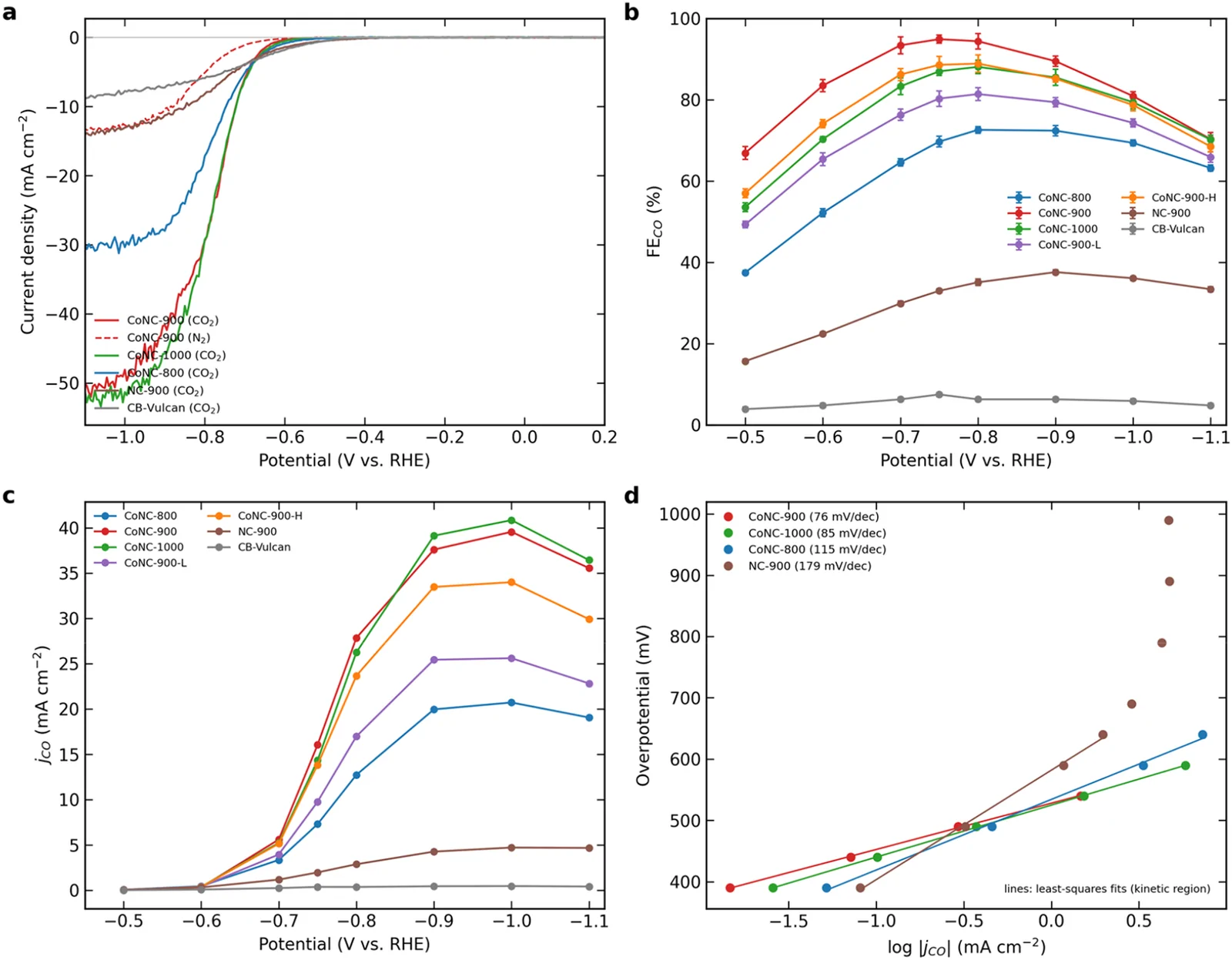
**(a)** LSV curves (5 mV s^−1^) in CO_2_- and N_2_-saturated 0.5 M KHCO_3_; CoNC-900-L and CoNC-900-H are omitted for clarity, the N_2_ control is shown for CoNC-900, and N_2_ controls for all seven samples are given in Supplementary Figure S8. **(b)** FE_CO versus applied potential (error bars: standard deviation of three independently prepared electrodes, n = 3). **(c)** CO partial current densities. **(d)** Tafel plots derived from j_CO in the kinetically controlled region for CoNC-900, CoNC-1000, CoNC-800 and NC-900, with fitted slopes annotated; slopes for CoNC-900-L and CoNC-900-H are given in Table 2.

The maximum FE_CO follows the order CoNC-900 (94.9%) > CoNC-900-H (88.9%) > CoNC-1000 (88.1%) > CoNC-900-L (81.4%) > CoNC-800 (72.6%) ≫ NC-900 (37.6%) ≫ CB-Vulcan (7.5%). The dramatic gap between CoNC-900 and the Co-free NC-900—prepared by an otherwise identical route—demonstrates unambiguously that N-doped carbon alone is HER-dominated under these conditions (FE_H_2_ up to 83% on NC-900) and that the Co–N_x_ sites are the primary CO-producing centers. Two volcano-type relationships emerge from the controlled series. With respect to pyrolysis temperature, CoNC-800 suffers from poor conductivity (charge-transfer resistance R_ct = 118 Ω; Section 3.6) despite its high nitrogen content, whereas CoNC-1000 is the most conductive and most porous but has lost both nitrogen (5.2 at%) and Co–N_x_ sites; CoNC-900 optimally balances these opposing regimes. With respect to Co loading, 5 mol% feed provides too few active sites, while 20 mol% feed raises the bulk Co to 5.83 wt% and slightly increases H_2_ selectivity, consistent with incipient formation of acid-resistant Co aggregates; the 10 mol% feed maximizes accessible Co–N_x_ density without aggregation. A possible contribution of residual Zn–N_x_ sites, which are themselves CO-selective (Yang F. et al., 2018), can be excluded on the basis of the internal control: NC-900 contains residual Zn (0.29 wt%) at a level comparable to CoNC-900 (0.34 wt%) yet reaches only 37.6% FE_CO with 3.1 μA cm^−2^_ECSA intrinsic activity versus 18.9 μA cm^−2^_ECSA for CoNC-900 (Section 3.6); the six-fold higher intrinsic CO activity of CoNC-900 therefore cannot be accounted for by Zn and is attributable to the Co–N_x_ sites, with any Zn contribution bounded by the NC-900 baseline.

In order to put the activity on a site-normalized basis, we calculated conservative lower-bound turnover frequencies (TOF), based on the assumption that all of the Co atoms in the electrode are active sites. At −0.75 V, the loading of 0.51 mg cm^−2^ and bulk Co content of 2.91 wt% correspond to 2.52 × 10^−7^ mol Co cm^−2^, so j_CO = 16.0 mA cm^−2^ translates into TOF ≥0.33 s^−1^ (≈1.2 × 10^3^ h^−1^), a CO mass activity of 31.4 A g^−1^ of catalyst, and 1.08 kA g^−1^ of Co. Because only a fraction of the Co atoms is electrochemically accessible, the true per-site turnover frequency is necessarily higher than this bound.

Tafel analysis of the kinetically controlled region (Figure 6d) was performed by least-squares regression of overpotential against log j_CO. The fitted slopes are 76 mV dec^−1^ for CoNC-900 (R^2^ = 0.999), 85 mV dec^−1^ for CoNC-1000 (R^2^ = 1.000), 94 mV dec^−1^ for CoNC-900-H, 106 mV dec^−1^ for CoNC-900-L, and 115 mV dec^−1^ for CoNC-800 (R^2^ ≥ 0.996). NC-900 exhibits pronounced curvature across its overpotential range; its low-overpotential linear region gives ≈179 mV dec^−1^, and CB-Vulcan shows no defined Tafel behavior, consistent with an interface dominated by hydrogen evolution rather than CO_2_ activation. The value of 76 mV dec^−1^ for CoNC-900—intermediate between the 59 mV dec^−1^ expected for a fast pre-equilibrium electron transfer followed by a rate-limiting chemical step and the 118 mV dec^−1^ of a rate-determining first electron transfer—indicates fast initial electron transfer with the proton-coupled formation of *COOH controlling the overall rate, in agreement with mechanistic studies of M–N–C catalysts (Ju et al., 2019) and with the DFT results in Section 3.8. A full summary of the electrochemical descriptors is given in Table 2.

**TABLE 2:** Summary of electrochemical CO_2_RR performance in CO_2_-saturated 0.5 M KHCO_3_. Maximum FE_CO, j_CO at −0.75 V, Tafel slope, charge-transfer resistance (R_ct), and double-layer capacitance (C_dl).

Sample	Max FE_CO (%)	j_CO at −0.75 V (mA cm^−2^)	Tafel (mV dec^−1^)	R_ct (Ω)	C_dl (mF cm^−2^)
CoNC-900	94.9	16.0	76	44	33.9
CoNC-1000	88.1	14.3	85	40	35.6
CoNC-900-H	88.9	13.8	94	53	30.8
CoNC-900-L	81.4	9.8	106	64	29.0
CoNC-800	72.6	7.3	115	118	18.7
NC-900	37.6	2.0	≈179^a^	519	25.5
CB-Vulcan	7.5	0.4	n.d.^b^	1,200	8.4

Tafel slopes are least-squares fits of overpotential vs. log j_CO over the kinetic region (R^2^ ≥ 0.996 unless noted).

^a^ NC-900, shows pronounced curvature; the value refers to the low-overpotential linear region (first four points).

^b^ Not defined: CB-Vulcan j_CO is non-monotonic with overpotential, consistent with HER, domination.

### 3.6 Interfacial kinetics and active-site accessibility

The double-layer capacitance measurements (Figure 7a) have the same trend as the BET measurements, yielding C_dl values of 18.7, 33.9, and 35.6 mF cm^−2^ for CoNC-800, CoNC-900, and CoNC-1000 respectively (linear fits, R^2^ ≥ 0.999), which is in agreement with the BET trend and confirms the electrochemically wetted and accessible hierarchical pore network. However, the selectivity ranking is not just based on the surface area - CoNC-1000 has the highest ECSA, but lower FE_CO and j_CO than CoNC-900, and Co-free NC-900 has a moderately high ECSA but almost no CO activity. Across the controlled series, therefore, the density and quality of the Co–N_x_ sites determine the intrinsic activity, while the hierarchical porosity governs how much of that site population is electrochemically accessible.

**FIGURE 7:**
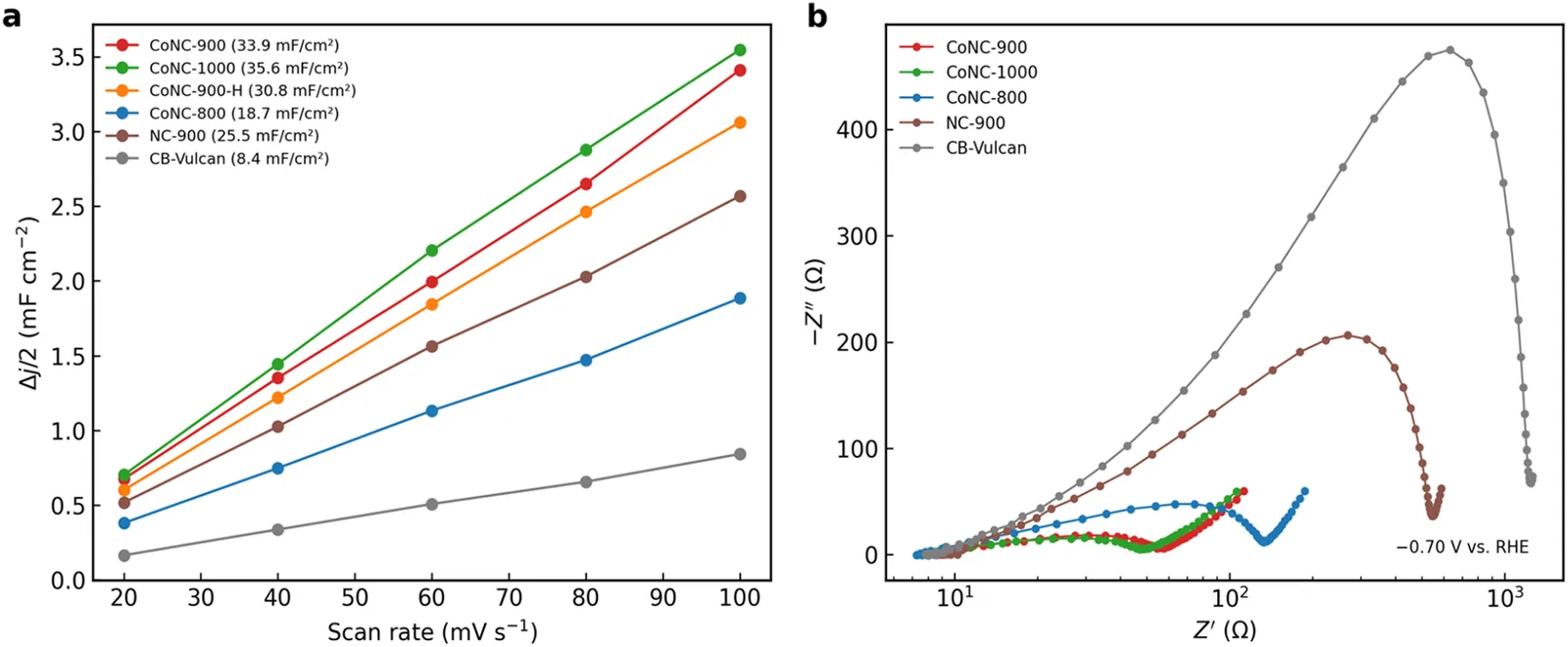
**(a)** Capacitive current difference (Δj/2) versus scan rate used to extract C_dl; slopes are proportional to the electrochemically active surface area. **(b)** Nyquist plots at −0.70 V vs. RHE (log scale) with equivalent-circuit fits (Rs + Q/(Rct + Zw)).

The interfacial charge-transfer kinetics are quantified by Nyquist plots (Figure 7b) recorded at a potential of −0.70 V vs. RHE. The fitted charge-transfer resistances are 44 Ω (CoNC-900) and 40 Ω (CoNC-1000) versus 118 Ω (CoNC-800), 519 Ω (NC-900), and 1,200 Ω (CB-Vulcan). The sharp decrease in R_ct from CoNC-800 to CoNC-900 demonstrates the conductivity enhancement that is obtained by the graphitization process; the roughly twelve-fold difference between CoNC-900 and NC-900 demonstrates the intrinsic kinetic superiority of the Co-containing interface. The C_dl and EIS data reveal that the combination of the large available surface, coupled with the rapid charge transfer at the abundant Co–N_x_ sites is the reason CoNC-900 is at the tip of both activity volcanoes. The catalysts are then put on an intrinsic-activity scale by normalizing j_CO at −0.75 V based on the ECSA-derived roughness factor. CoNC-900 delivers 18.9 μA cm^−2^_ECSA, exceeding CoNC-900-H (17.9), CoNC-1000 (16.1), CoNC-800 (15.6), and CoNC-900-L (13.5 μA cm^−2^_ECSA), while the Co-free NC-900 reaches only 3.1 μA cm^−2^_ECSA. The activity of CoNC-900 is therefore rooted in the density and quality of the Co–N_x_ sites rather than in surface area alone. Because C_s enters every ECSA value as a common divisor, the intrinsic-activity ranking is independent of the specific value adopted for C_s; only the absolute μA cm^−2^_ECSA numbers would scale, so the conclusions drawn from the ECSA-normalized comparison are insensitive to this choice.

### 3.7 Stability

The durability of CoNC-900 was tested by 24 h chronoamperometry (Figure 8) under −0.75 V vs. RHE. The current density decreases gradually from −16.1 mA cm^−2^ (on an average over the first and last 10 min) to −14.3 mA cm^−2^ (≈11% loss), whereas FE_CO, which was sampled every 4 h, maintains above 92%. Since there was no sudden selectivity drop, there is no significant evidence of large-scale Co restructuring or leaching, and the slight decrease in selectivity at present is due to the common H-cell artifacts (partial electrode flooding, pH drift in unrefreshed electrolyte) that occur under normal operating conditions. This is compared favorably with the stability of a number of reported H-cell Co–N–C catalysts (Table 3), and further evidences the robustness of the acid-etched Co–N_x_ sites anchored in the graphitized matrix. Extended operation in gas-diffusion or membrane-electrode configurations, which alleviate flooding and CO_2_ starvation, is a natural avenue for further durability testing.

**FIGURE 8:**
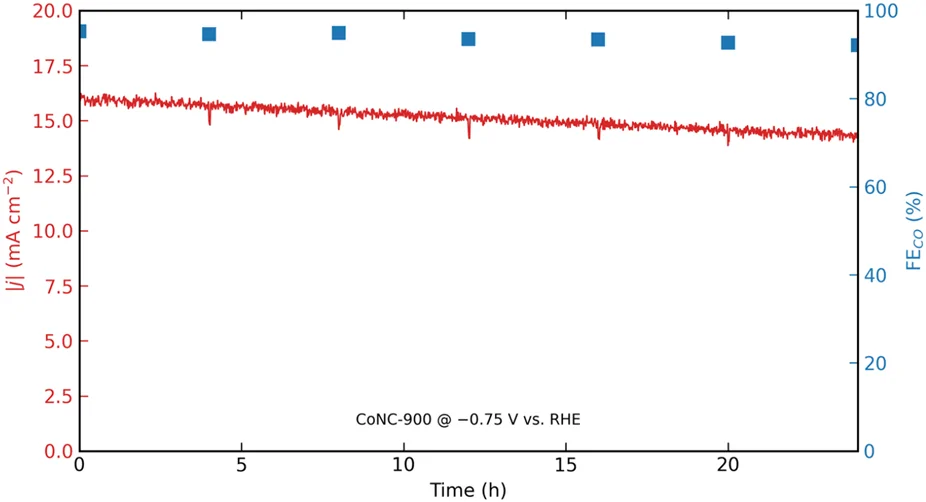
Chronoamperometric stability of CoNC-900 at −0.75 V vs. RHE for 24 h in CO_2_-saturated 0.5 M KHCO_3_ (red line, current density; blue squares, FE_CO sampled every 4 h).

**TABLE 3:** Comparison of CO_2_-to-CO performance of CoNC-900 with representative reported catalysts. Values are as reported by the original authors; cell type is indicated because gas-diffusion/flow cells generally enable higher current densities than H-cells.

Catalyst	FE_CO	j_CO (mA cm^−2^)	Conditions	Cell	Ref.
CoNC-900 (this work)	94.9% ± 1.0%	16.0	−0.75 V, 0.5 M KHCO_3_; 24 h stable	H-cell	—
NC-900 (this work, Co-free)	37.6%	2.0	−0.75 V, 0.5 M KHCO_3_	H-cell	—
Co–N_5_/HNPCS	>99%	—	−0.73/−0.79 V; >90% over −0.57 to −0.88 V; 10 h	H-cell	Pan Y. et al. (2018)
Co–N_2_ single sites	94%	18.1	η = 520 mV; TOF 18,200 h^−1^	H-cell	Wang et al. (2018)
Ni–N_4_ (exclusive sites)	near-unity	—	0.5 M KHCO_3_	H-cell	Li et al. (2017)
Ni single sites (MOF ion exchange)	71.9%	10.48	η = 0.89 V; TOF 5273 h^−1^	H-cell	Zhao et al. (2017)
Ni(I) single atoms	97%	up to 22	η = 0.61 V; 100 h stable	H-cell	Yang H. B. et al. (2018)
NiSA–N_2_–C (MOF-templated)	98%	—	TOF 1622 h^−1^	H-cell	Gong et al. (2020)
Ni–N–C (GDE electrolyzer)	≈85%	>200	up to 700 mA cm ^−2^ total	GDE flow	Möller et al., (2019)
CoPc (molecular, high rate)	—	up to 165	aqueous, GDE-type operation	GDE flow	Wang et al. (2019)
CoPc-CN/CNT hybrid	>95%	15.0	η = 0.52 V; TOF 4.1 s^−1^	H-cell	Zhang et al. (2017)
Co-porphyrin COF	90%	—	η = 0.55 V; TOF 3 s^−1^	H-cell	Lin et al. (2015)

All literature values were verified against the abstracts of the original publications; — indicates the quantity is not stated in the source abstract, and ≈ denotes a value reported as approximate by the original authors. Gas-diffusion (GDE) entries relieve CO_2_ mass-transport limits and are not directly comparable to H-cell data.

### 3.8 Mechanistic insight from DFT

The CO_2_ → COOH → CO → CO(g) pathway was calculated on Co–N_4_, pyridinic-N, graphitic-N and pristine-carbon models to rationalize the site-specific selectivity (Figure 9a). The potential limiting step is the first proton coupled electron transfer to form COOH, whose free energy barrier is vastly different on Co–N_4_, 0.42 eV, compared to the other metal-free sites, 1.79 eV, 2.24 eV, and 3.15 eV at U = 0 V, and this point directly explains why the FE_CO of NC-900 is only 37.6% whereas it is nearly 95% for CoNC-900. Again, the CO adsorption energy on Co–N_4_ is not particularly high (−0.72 eV), which explains why CO desorption is only slightly endothermic, in contrast to the relatively strongly CO-bound metals, which exhibit the site poisoning that would be expected given their shallower d-band center −1.17 eV compared with −1.35 eV for Co–N_4_).

**FIGURE 9:**
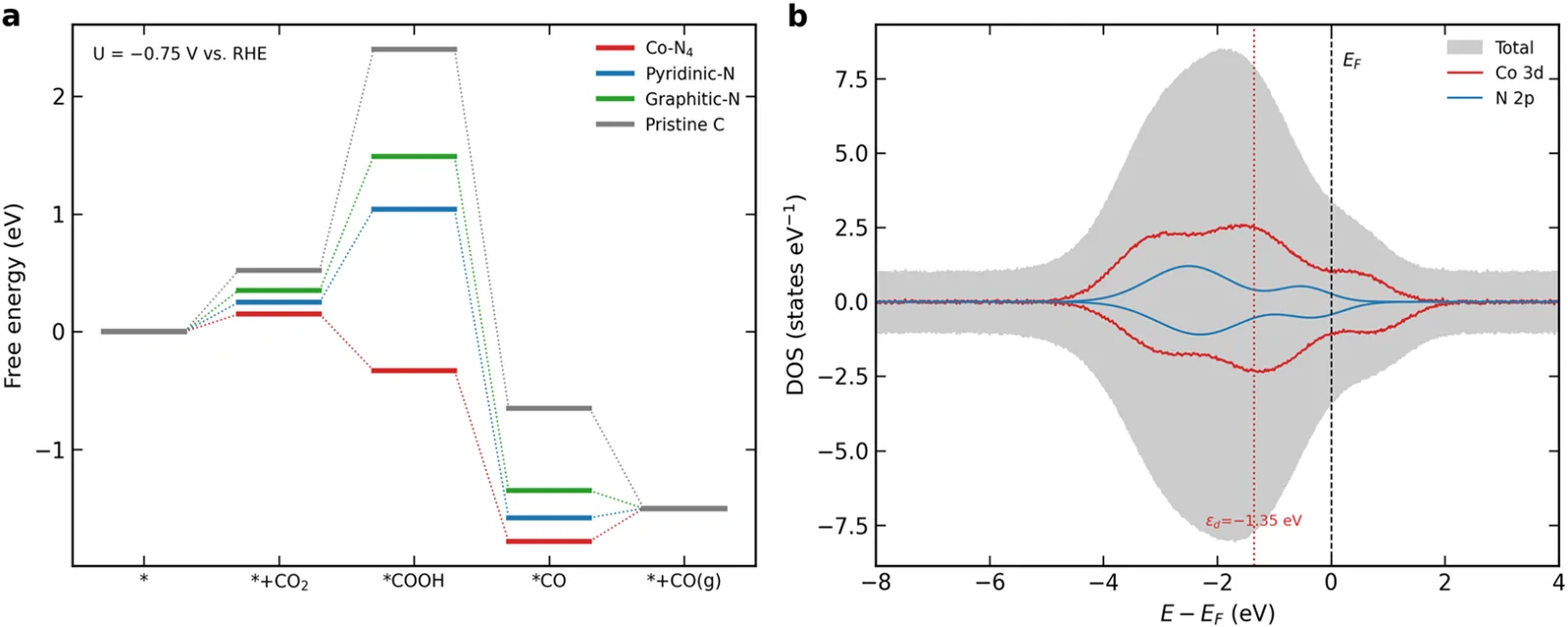
**(a)** Free-energy diagrams for CO_2_ reduction to CO on Co–N_4_, pyridinic-N, graphitic-N, and pristine-carbon sites at U = −0.75 V vs. RHE (CHE model with implicit solvation). **(b)** Total and projected (Co 3d, N 2p) densities of states of the Co–N_4_ model; the Co 3d band center (−1.35 eV) and Fermi level are indicated.

The competing HER completes the selectivity picture. Bader charge analysis reveals a substantial transfer of 0.38 e to the adsorbed *COOH intermediate on Co–N_4_, together with electron accumulation between the Co centre and the carboxyl carbon, identifying Co as the electron-donating anchor that stabilizes the bent CO_2_-derived intermediate. Pyridinic-N, in contrast, has a stronger binding energy of ΔE_ads(H) = −0.45 eV, than Co–N_4_ (−0.32 eV), and COOH is not well stabilized on the metal-free sites, making metal-free N-doped carbon intrinsically biased toward HER, as found for NC-900. The model Co–N_4_ (Figure 9b) predicts a density of states with the center of the Co 3d band at −1.35 eV, inside the range corresponding to medium-strength binding, which is a compromise between COOH stabilization and CO release. This d-band picture gives rise to a transferable descriptor: the CO selectivity of M–N–C catalysts can be modified by tuning the d-band center of the metal by changing the metal and the number of N coordinating to the metal, which is reminiscent of the superior performance of the coordination-engineered Co–N_x_ motifs reported by others (Pan Y. et al., 2018; Wang et al., 2018).

### 3.9 Benchmarking against reported catalysts

Table 3 compares the activity of CoNC-900 with various representative M–N–C, molecular and reference catalysts under similar conditions. CoNC-900 has the best performance of all the ZIF-derived M–N–C catalysts in the aqueous KHCO_3_ cell, with higher j_CO than almost all of the other literature values for H cells at similar selectivity (Gong et al., 2020; Pan Y. et al., 2018; Wang et al., 2018), together with a fitted Tafel slope of 76 mV dec^−1^ and 24 h of continuous operation. The absolute current density of molecular and flow-cell systems such as cobalt phthalocyanine catalysts and Ni–N–C powders in gas-diffusion configurations are higher, as would be expected for the superior CO_2_ mass transport in gas-fed architectures (Möller et al., 2019; Wang et al., 2019); immobilized cobalt porphyrins provide the molecular analogy for the Co–N_x_ motif (Hu et al., 2017; Lin et al., 2015). These comparisons indicate that the present precursor-level strategy—bimetallic Zn/Co dilution combined with DCD-assisted nitrogen retention—delivers site quality and accessibility competitive with far more elaborate synthetic routes, and situates it within a field that is advancing rapidly toward industrially relevant current densities in gas-fed architectures: lanthanide single-atom catalysts now sustain ≥90% FE_CO at 500 mA cm^−2^ (Wang et al., 2025), durable proton-exchange-membrane CO_2_ electrolyzers have been demonstrated (Fang et al., 2024), and systematic cell-level design rules for CO_2_-to-CO electrolyzers are emerging (Brückner et al., 2024), defining the performance targets toward which powder catalysts such as CoNC-900 must ultimately be developed. A summary performance overview and the pyrolysis-temperature volcano are provided in Supplementary Figure S7 (Supplementary Material).

## 4 Conclusion

In summary, a family of hierarchically porous Co–N–C catalysts bearing highly dispersed Co–N_x_ sites was synthesized through a simple, template-free route that combines a bimetallic Zn/Co-ZIF precursor, dicyandiamide co-pyrolysis, and acid etching, and was interrogated through a seven-sample controlled matrix spanning pyrolysis temperature (800 °C–1,000 °C), Co feed ratio (5–20 mol%), and metal-free and non-templated references. The dual role of Zn—as a Co-diluting matrix during crystallization and a self-sacrificial porogen during pyrolysis—together with DCD-assisted nitrogen retention (8.5 at% N at 900 °C) yields a carbon that unites a high surface area (1,083 m^2^ g^−1^, 47% mesopore volume), strong CO_2_ affinity (3.51 mmol g^−1^ at 273 K), and abundant Co^2+^–N_x_ coordination sites supported by converging XRD, XPS, EDS-mapping, and acid-stability evidence. The optimized CoNC-900 delivers a CO Faradaic efficiency of 94.9% ± 1.0% with a CO partial current density of 16.0 mA cm^−2^ at −0.75 V vs. RHE, a fitted Tafel slope of 76 mV dec^−1^, a charge-transfer resistance of 44 Ω, a lower-bound turnover frequency of 0.33 s^−1^ (31.4 A g^−1^ mass activity), and ≈11% current decay with FE_CO retained above 92% over 24 h of continuous electrolysis.

Mechanistically, the controlled series resolves two volcano relationships that are usually confounded. Along the temperature axis, graphitization (I_D/I_G from 1.15 to 0.97; R_ct from 118 to 40 Ω) is purchased at the cost of nitrogen retention, which falls from 12.8 to 5.2 at%, and of Co–N_x_ speciation, which falls from 18.5% to 14.2% of the N 1s signal, with 900 °C marking the optimum. Along the loading axis, 10 mol% Co maximizes the accessible Co–N_x_ density, while aggregation and rising H_2_ selectivity set in at 20 mol%. Normalization by the electrochemically active surface area shows that CoNC-900 remains the most active catalyst on an intrinsic footing (18.9 μA cm^−2^_ECSA versus 3.1 μA cm^−2^_ECSA for the Co-free control), demonstrating that the selectivity ranking is governed by site density and quality rather than by texture. DFT calculations complete the picture: *COOH formation is the potential-limiting step on every site examined, and only the Co–N_4_ moiety lowers its free-energy barrier into a catalytically relevant window (0.42 eV, becoming exergonic at −0.75 V), while its moderate *CO binding energy (−0.72 eV) averts site poisoning—a computed pathway that is quantitatively consistent with the measured 76 mV dec^−1^ Tafel slope and with the HER-dominated behaviour of the metal-free nitrogen sites.

Three transferable design rules emerge: dilute the active metal within a volatile-metal framework to enforce dispersion; supply an external nitrogen reservoir during carbonization to preserve the coordinating environment; and pyrolyze just above the porogen boiling point to balance conductivity against site retention. These rules are agnostic to the identity of the active metal and should extend to Fe-, Ni-, and dual-metal M–N_x_ systems. The modularity of the precursor-level strategy presented here—in which pore architecture, nitrogen chemistry, and active-site formation are all encoded before pyrolysis begins—makes it a practical platform for the development of earth-abundant CO_2_ reduction catalysts.

## Data Availability

The original contributions presented in the study are included in the article/Supplementary Material, further inquiries can be directed to the corresponding author.
