# Supplementary material for: Hierarchically porous Co–N–C catalysts from bimetallic Zn/Co zeolitic imidazolate frameworks for selective CO2 electroreduction to CO
Source: Front Chem. 2026 Sep 17;14:1940515. doi: 10.3389/fchem.2026.1940515 (PMC13627418; doi:10.3389/fchem.2026.1940515)
Supplement: Supplementary file 1 [file DataSheet1.pdf]

Supporting Information

Hierarchically Porous Co–N–C Catalysts from Bimetallic Zn/Co Zeolitic Imidazolate Frameworks for Selective CO_2 Electroreduction to CO

[Author 1][1], [Author 2][1], [Corresponding Author][1]*

Contents

Figures S1–S7: XPS survey spectra; capacitance voltammograms; CO_2 adsorption at 298 K; ^{1}H NMR of the catholyte; GC calibration curves; H_2 Faradaic efficiencies; performance summary and pyrolysis-temperature volcano. Tables S1–S5: EIS equivalent-circuit fitting parameters; N 1s deconvolution parameters; ICP-OES metal contents; EDS quantification; double-layer capacitance and ECSA.

Supporting Figures

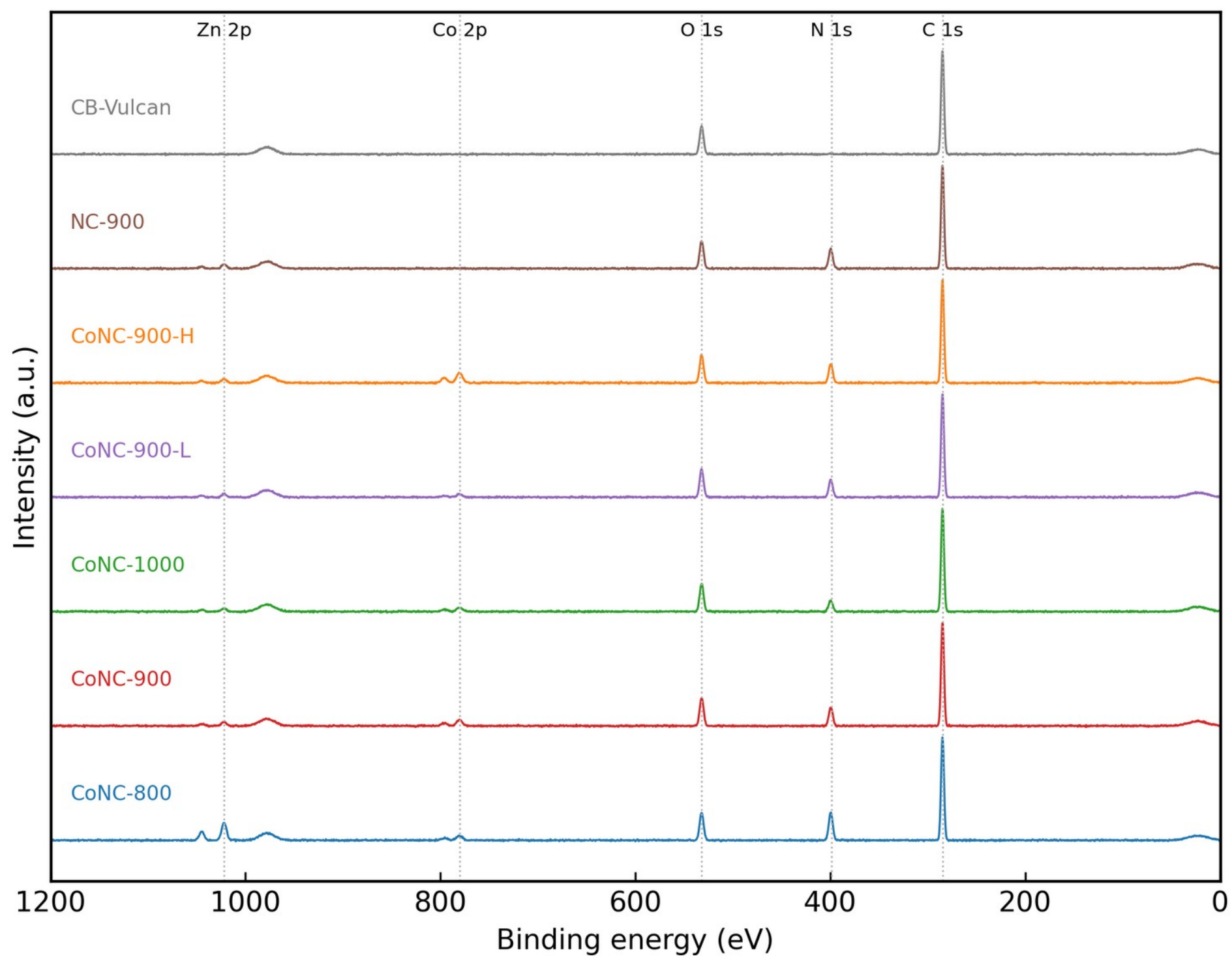

Figure S1. XPS survey spectra of all seven samples. Principal core levels (C 1s, N 1s, O 1s, Co 2p, Zn 2p) are indicated. The Zn 2p intensity decreases sharply with pyrolysis temperature, consistent with Zn volatilization above 907 °C, and Co 2p is absent in NC-900 and CB-Vulcan.

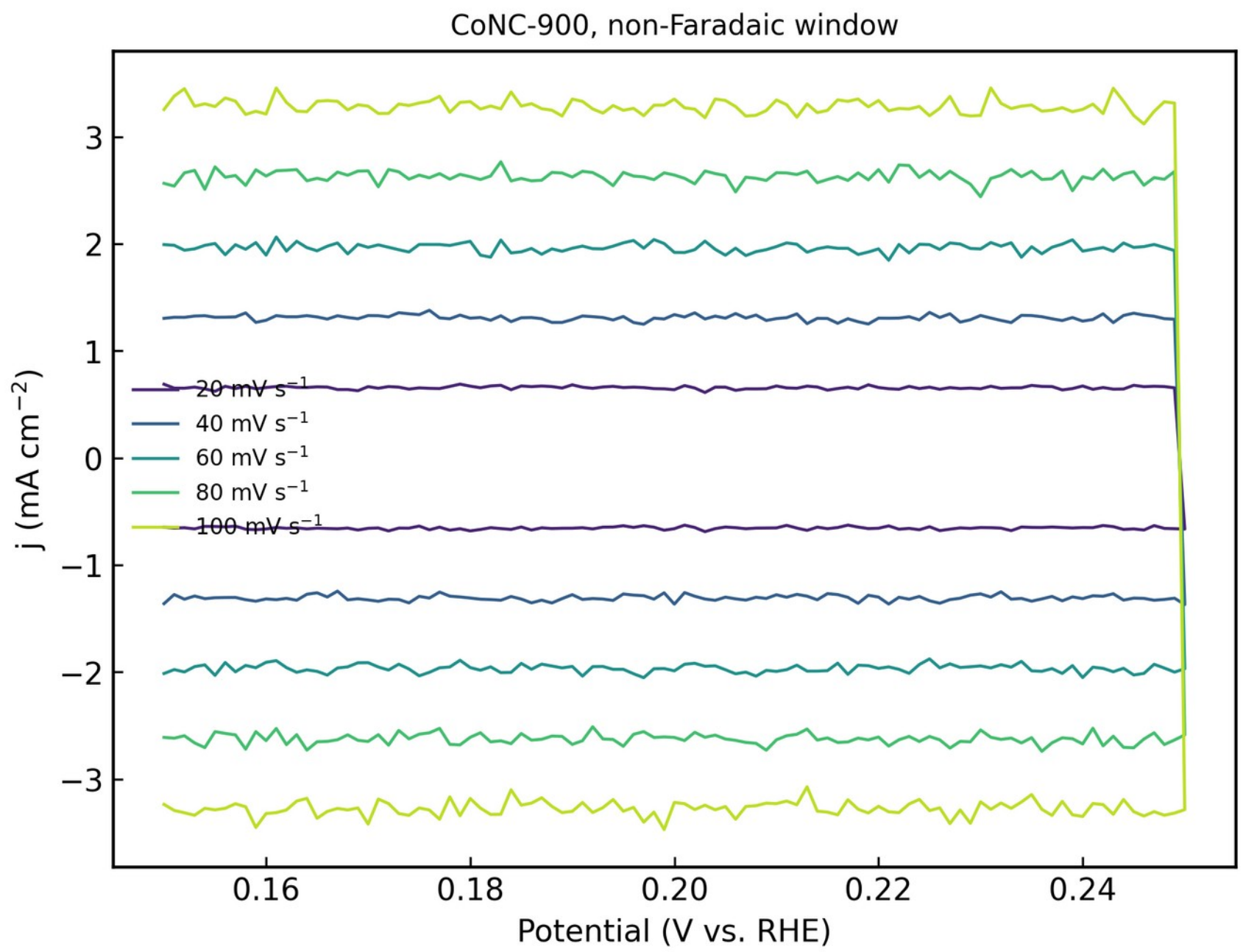

Figure S2. Cyclic voltammograms of CoNC-900 in the non-Faradaic window (0.15–0.25 V vs. RHE) at 20–100 mV s^{-1}, used for double-layer capacitance extraction. Equivalent datasets were collected for all samples (Table S5).

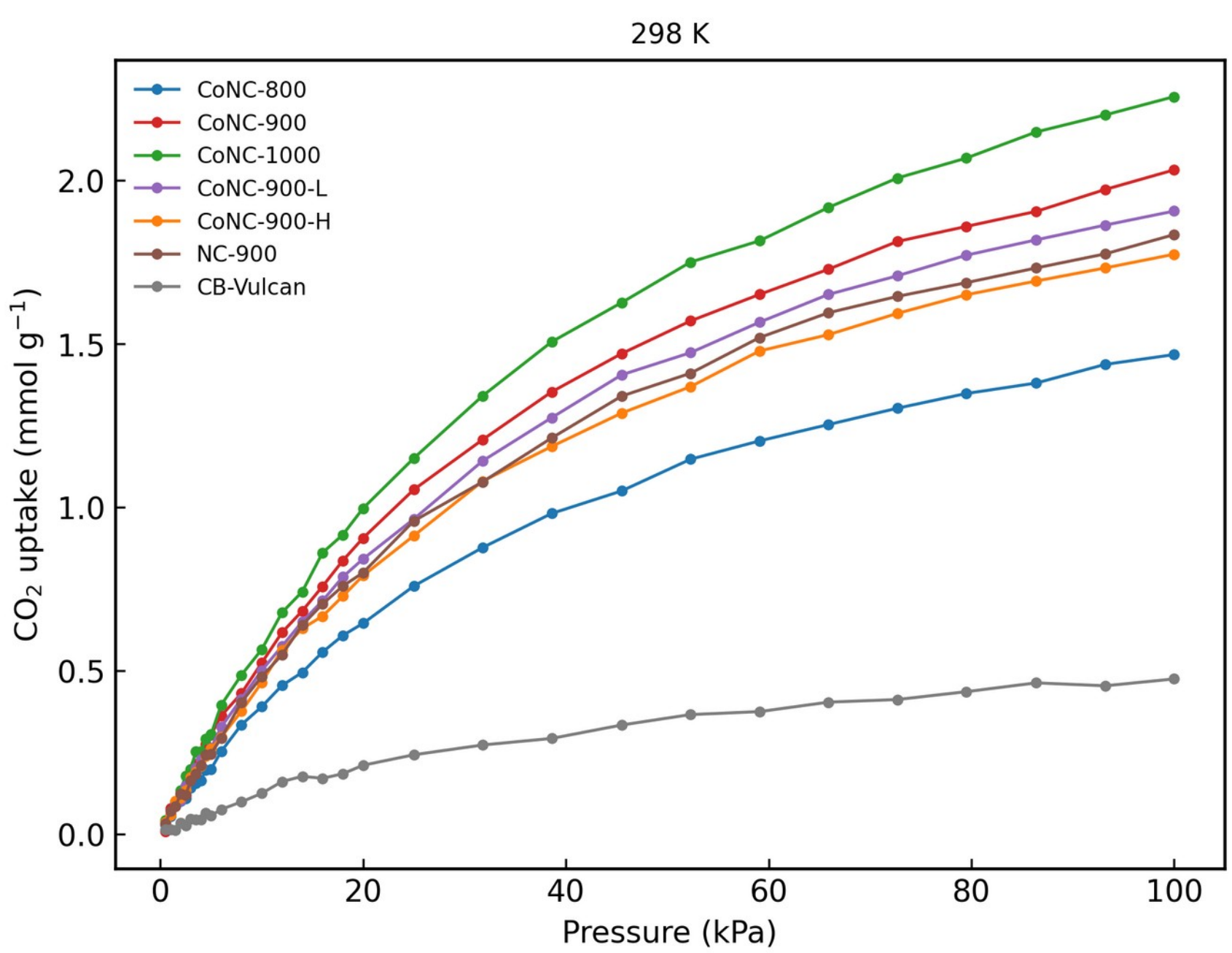

Figure S3. CO_2 adsorption isotherms at 298 K for all samples, complementing the 273 K data in Figure 4c of the main text. The sample ordering is preserved at both temperatures.

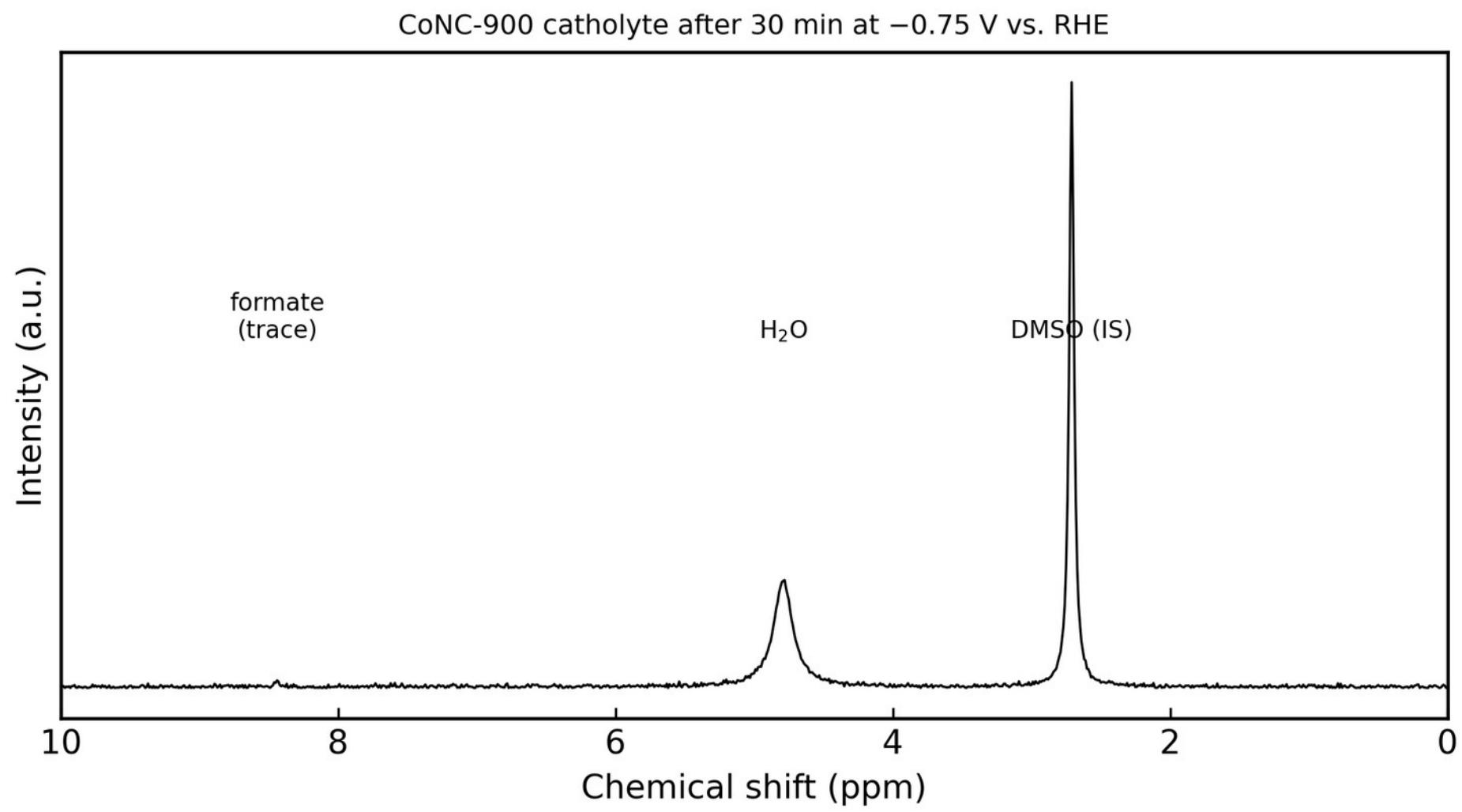

Figure S4. ¹H NMR spectrum (400 MHz, WATERGATE water suppression, 512 scans) of the CoNC-900 catholyte after 30 min of electrolysis at −0.75 V vs. RHE, with DMSO as internal standard. Only trace formate is detected (0.032 ± 0.005 mM, FE < 1.5%); methanol, ethanol, and acetate are below their detection limits (0.01–0.02 mM).

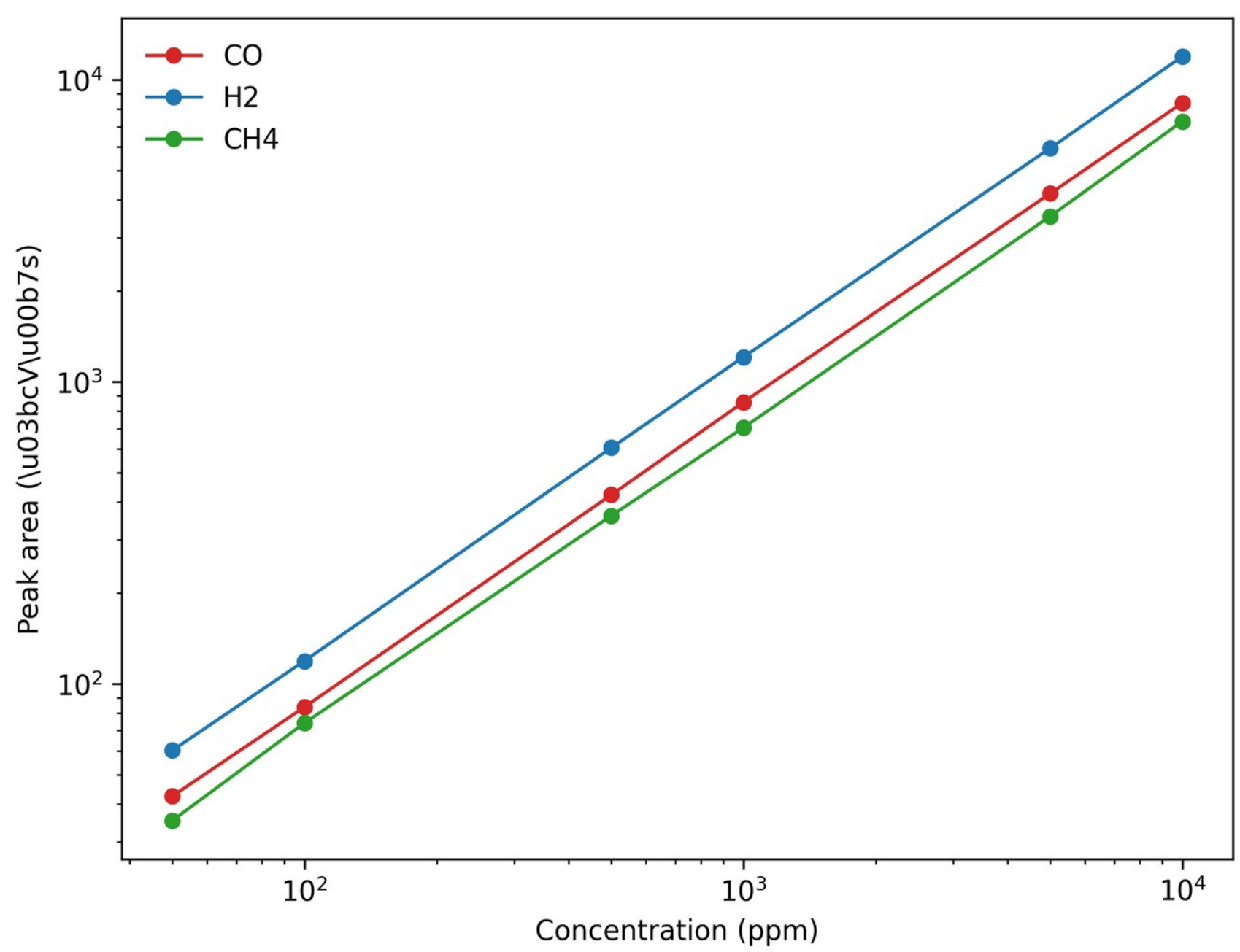

Figure S5. Six-point GC calibration curves for CO, H_2, and CH_4 (50–10,000 ppm) used for Faradaic-efficiency quantification (Agilent 7890B; TCD for H_2/CO, FID with methanizer for hydrocarbons).

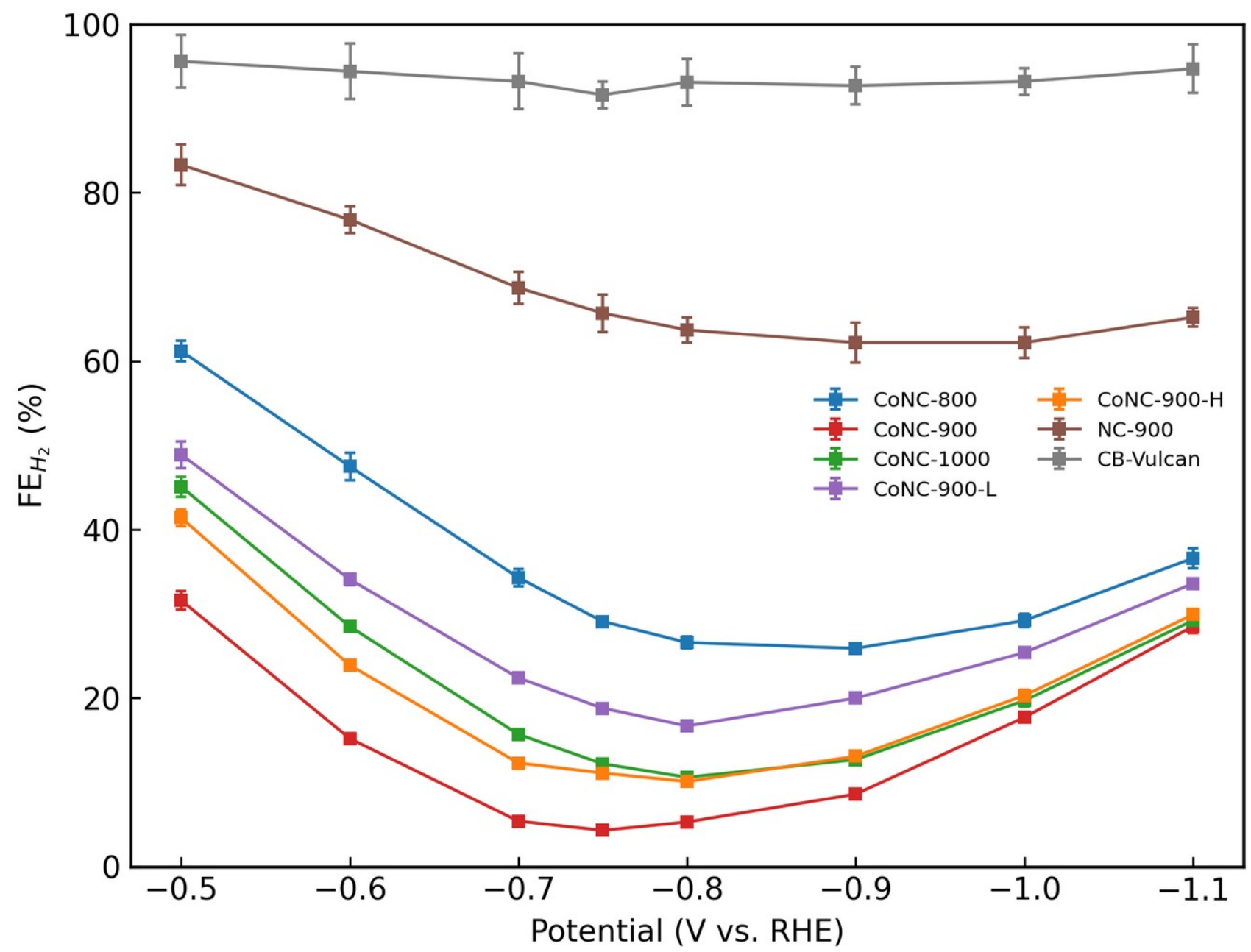

Figure S6. Potential-dependent H_2 Faradaic efficiencies of all samples (error bars: standard deviation, n = 3). FE_H_2 mirrors FE_CO such that the total Faradaic efficiency closes to 99–100% at every potential.

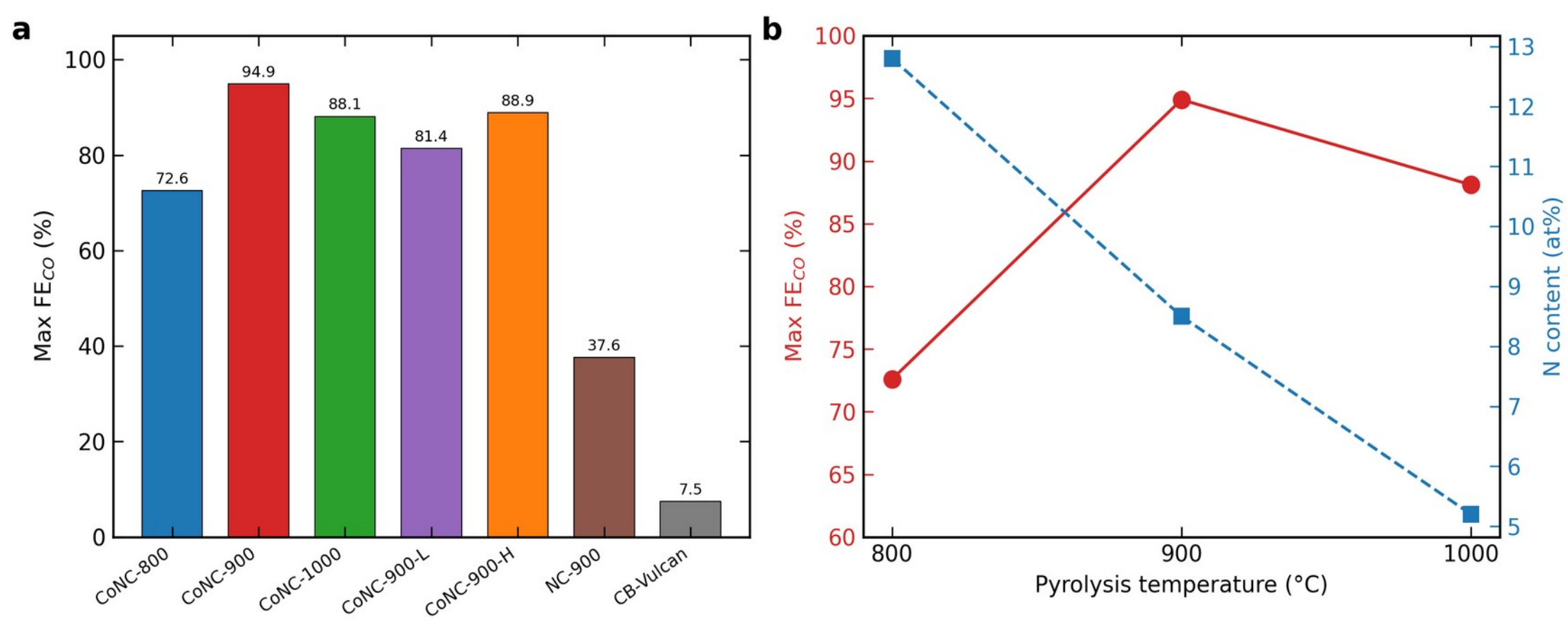

Figure S7. (a) Maximum FE_CO across the seven catalysts. (b) Volcano relationship for the 10 mol% Co temperature series: FE_CO (red) peaks at 900 °C where nitrogen retention (blue) and graphitization/conductivity are balanced.

Supporting Tables

Table S1. EIS equivalent-circuit fitting parameters at −0.70 V vs. RHE (circuit: Rs + Q/(Rct + Zw); ZView; $\chi^2 < 0.01$ for all fits).

Sample	Rs (Ω)	Rct (Ω)	Q (F s^{n-1})	n	σw (Ω $s^{-0.5}$)	χ^2
CoNC-800	7.7	118.1	6.10×10^{-5}	0.863	15.8	0.0045
CoNC-900	7.8	43.6	9.75×10^{-5}	0.852	15.9	0.0060

Sample	Rs (Ω)	Rct (Ω)	Q (F s^{n-1})	n	σw (Ω $s^{-0.5}$)	χ^2
CoNC-1000	8.1	39.8	1.10×10^{-4}	0.852	15.4	0.0045
CoNC-900-L	8.0	64.4	8.95×10^{-5}	0.831	13.5	0.0023
CoNC-900-H	7.6	53.4	9.38×10^{-5}	0.849	13.4	0.0049
NC-900	8.4	518.7	7.65×10^{-5}	0.846	15.5	0.0058
CB-Vulcan	7.6	1199.5	2.63×10^{-5}	0.850	16.4	0.0060

Table S2. N 1s deconvolution parameters (Shirley background, GL30 line shape, charge-corrected to C 1s = 284.8 eV).

Sample	Pyridinic-N (398.5 eV) %	Co–N_x (399.2–399.3 eV) %	Pyrrolic-N (400.3 eV) %	Graphitic-N (401.2 eV) %	Oxidized-N (403.5 eV) %
CoNC-800	32.1	15.2	20.5	24.8	7.4
CoNC-900	35.2	18.5	15.8	24.3	6.2
CoNC-1000	28.5	14.2	12.3	35.8	9.2
CoNC-900-L	34.8	9.5	17.2	31.5	7.0
CoNC-900-H	30.5	25.8	14.2	22.5	7.0
NC-900	38.5	—	18.5	33.8	9.2
CB-Vulcan	35.0	—	—	65.0	—

Table S3. Bulk metal contents by ICP-OES (aqua-regia microwave digestion; detection limits: Co 0.005, Zn 0.008, Fe 0.003, Ni 0.004 ppm).

Sample	Co (wt%)	Zn (wt%)	Fe (wt%)	Ni (wt%)
CoNC-800	3.82	2.15	<0.01	<0.01
CoNC-900	2.91	0.34	<0.01	<0.01
CoNC-1000	2.38	0.11	<0.01	<0.01
CoNC-900-L	1.48	0.31	<0.01	<0.01
CoNC-900-H	5.83	0.37	<0.01	<0.01
NC-900	<0.01	0.29	<0.01	<0.01
CB-Vulcan	<0.01	<0.01	<0.01	<0.01

Table S4. Surface composition by EDS (15 kV, three-spot average per sample).

Sample	C (at%)	N (at%)	O (at%)	Co (at%)	Zn (at%)
CoNC-800	81.3	10.3	4.6	1.9	1.9
CoNC-900	85.7	6.2	5.0	2.6	0.5
CoNC-1000	88.1	4.4	5.4	1.8	0.3
CoNC-900-L	87.1	6.4	5.1	1.1	0.4
CoNC-900-H	83.3	6.8	5.0	4.5	0.4
NC-900	87.5	6.9	5.1	0.0	0.5
CB-Vulcan	95.6	0.0	4.4	0.1	0.0

Table S5. Double-layer capacitance and estimated ECSA. C_dl values were obtained by ordinary least-squares regression of Δj/2 (0.20 V vs. RHE) against scan rate over 20–100 mV s^{-1} from the raw voltammograms; ECSA = C_dl/C_s with C_s = 0.040 mF cm^{-2}.

Sample	C_dl (mF cm^{-2})	R^2 of fit	ECSA (cm^2)
CoNC-800	18.7	0.9993	468
CoNC-900	33.9	0.9991	848
CoNC-1000	35.6	0.9991	890
CoNC-900-L	29.0	0.9999	725
CoNC-900-H	30.8	0.9999	770
NC-900	25.5	0.9996	638
CB-Vulcan	8.4	0.9992	210

Supporting Note S1. Faradaic efficiency calculation

Faradaic efficiencies were calculated as FE = nFN_product/Q_total, where n = 2 electrons per CO or H_2 molecule, F = 96,485 C mol^{-1}, N_product is the molar amount of product determined from the calibrated GC peak areas (Figure S5) and the known headspace volume and flow, and Q_total is the charge integrated over the 30 min potentiostatic step. All FE values are means of three independent electrodes (n = 3), with the standard deviation reported as the error.

Supporting Note S2. Lower-bound turnover frequency

TOF was estimated as TOF = j_CO/(2F·n_Co), where n_Co = m_loading × w_Co/M_Co is the areal molar amount of Co (0.51 mg cm^{-2} × 2.91 wt% / 58.93 g mol^{-1} = 2.52 × 10^{-7} mol cm^{-2} for CoNC-900). Because this assumes every Co atom in the film is an accessible active site, the value (0.33 s^{-1} at −0.75 V) is a strict lower bound on the per-site turnover frequency.

Supporting Information — Revision 1 additions

The following items are added to the Supporting Information in response to the reviewers. Figure S8 and Tables S6–S8 are new; Figures S1–S7 and Tables S1–S5 are unchanged from the original submission.

Figure S8

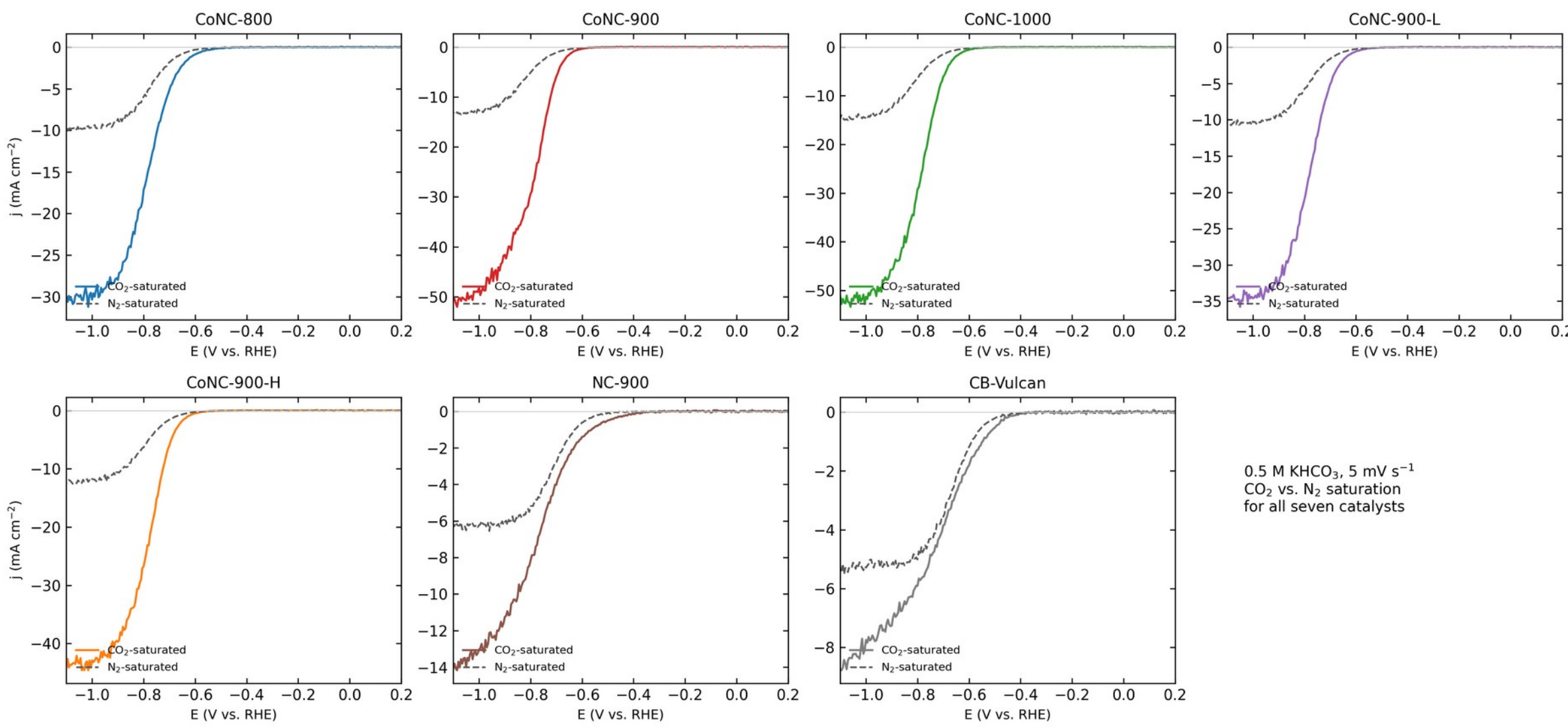

Figure S8. Linear sweep voltammograms (5 mV s^{-1}) of all seven catalysts in CO_2-saturated (colored solid lines) and N_2-saturated (grey dashed lines) 0.5 M $KHCO_3$. At −0.75 V vs. RHE the N_2-saturated current amounts to 15% (CoNC-900), 20% (CoNC-1000), 22% (CoNC-900-H), 27% (CoNC-900-L), and 35% (CoNC-800) of the corresponding CO_2-saturated current, compared with 69% for the Co-free NC-900 and 89% for CB-Vulcan, confirming that the CO_2-dependent current on the Co-containing catalysts arises from CO_2 reduction rather than hydrogen evolution.

Table S6

Table S6. Tafel fitting windows and goodness of fit. Overpotential η is referenced to E°(CO_2/CO) = −0.11 V vs. RHE. Fits are least-squares regressions of η against log j_CO over the kinetically controlled region, defined as potentials preceding the onset of curvature in the Tafel plot and at which j_CO remained below 30% of the diffusion-limited current estimated from the LSV plateau.

Sample	η window (mV)	Points (n)	Slope (mV dec^{-1})	R^2	Note
CoNC-900	390–540	4	76	0.999	—
CoNC-1000	390–590	5	85	1.000	—
CoNC-900-H	490–640	3	94	0.996	—
CoNC-900-L	490–640	3	106	0.997	—
CoNC-800	390–640	4	115	0.997	—
NC-900	390–640	4	179	0.998	low-η linear region; full-range fit (390–990 mV,

Sample	η window (mV)	Points (n)	Slope (mV dec⁻¹)	R^2	Note
					n = 8) gives 284 mV dec⁻¹ with R^2 = 0.82 owing to pronounced curvature
CB-Vulcan	—	—	n.d.	—	j_CO non-monotonic with η; no defined Tafel region

Table S7

Table S7. Crystallographic and Raman structural parameters. d_{002} and stacking height Lc from Scherrer analysis of the (002) reflection (K = 0.89, λ = 1.5406 Å; number of stacked layers N = Lc/d_{002}). In-plane crystallite size La from I_D/I_G using the Cançado relation La (nm) = $(2.4 \times 10^{-10}) \lambda^4 (I_D/I_G)^{-1}$ with λ = 532 nm [49].

Sample	(002) position (°2θ)	(002) FWHM (°2θ)	d_{002} (Å)	Lc (nm)	N layers	≈44° band centre (°2θ)	I_D/I_G	La (nm)
CoNC-800	22.8	9.9	3.89	8.1	21	43.0	1.15	16.7
CoNC-900	24.4	7.5	3.65	10.8	30	43.5	1.05	18.3
CoNC-1000	25.8	5.0	3.45	16.2	47	43.9	0.97	19.8
CoNC-900-L	24.9	7.4	3.58	10.8	30	43.9	1.06	18.1
CoNC-900-H	24.9	7.4	3.57	10.8	30	43.9	1.04	18.5
NC-900	24.9	7.4	3.58	10.9	31	43.7	1.08	17.8
CB-Vulcan	25.1	4.5	3.55	18.0	51	43.5	1.35	14.2

Table S8

Table S8. Quantitative Co 2p XPS parameters for the Co-containing catalysts (Shirley background; positions from smoothed spectra; FWHM of the $2p_3/_2$ main line; SOS = spin–orbit splitting $2p_1/_2 - 2p_3/_2$; ΔE_sat = satellite − main-line separation; I_sat/I_main = satellite-to-main intensity ratio). Reference values [52]: metallic Co $2p_3/_2$ 778.1–778.3 eV (asymmetric, no strong satellite); Co_3O_4 ≈779.6 eV with weak satellite ≈9 eV above; high-spin Co^{2+} 780–781 eV with intense satellite 5–6 eV above the main line.

Sample	$2p_3/_2$ (eV)	FWHM (eV)	$2p_1/_2$ (eV)	SOS (eV)	Satellite (eV)	ΔE_sat (eV)	I_sat/ I_main	Component at 778.2 eV
CoNC-800	780.0	2.5	795.8	15.8	785.5	5.5	0.37	none required
CoNC-900	780.3	2.5	795.8	15.5	785.1	4.9	0.34	none required
CoNC-1000	780.2	2.4	795.9	15.6	785.5	5.2	0.38	none required
CoNC-900-L	780.0	2.5	796.1	16.1	785.3	5.2	0.36	none required
CoNC-900-H	780.2	2.5	795.9	15.6	785.5	5.2	0.35	none required
